# Structure-Guide Design and Optimization of Potential Druglikeness Inhibitors for TGFβRI with the Pyrrolopyrimidine Scaffold

**DOI:** 10.3390/ph15101264

**Published:** 2022-10-13

**Authors:** Dan Meng, Jiali Xie, Yihao Li, Ruoyu Li, Hui Zhou, Ping Deng

**Affiliations:** 1 College of Pharmacy, Chongqing Medical University, Chongqing 400016, China; mengdan429@stu.cqmu.edu.cn (D.M.); xiejiali@stu.cqmu.edu.cn (J.X.); lyh1620885216@hotmail.com (Y.L.); lry@stu.cqmu.edu.cn (R.L.); 2 Chongqing Research Center for Pharmaceutical Engineering, Chongqing 400016, China; 3 Chongqing Key Research Laboratory for Quality Evaluation and Safety Research of APIs, Chongqing 400016, China

**Keywords:** TGFβRI, structural modification, molecular docking and dynamics, ADMET prediction, synthesis, bioactivity validation

## Abstract

Among all types of TGFβ signal blockers, small molecule kinase inhibitors (SMKIs) have attracted wide attention due to their economical production, obvious stability, and ease of oral administration. Nevertheless, SMKIs of TGFβRItypically have low druggability so there are none on the market. In this study, structure-based drug design (SBDD) was performed focusing on the pyrrolopyrimidin scaffold of BMS22 to find TGFβRIinhibitors with excellent medical potential. The binding mode, druggability, and target affinity were assessed by molecular docking, ADMET predictions, and molecular dynamics (MD) simulations for the designed TGFβRIinhibitors. Finally, the highly druggable compound W8 was discovered and then synthesized, which inhibited TGFβRIwith an IC_50_ value of about 10 μM. In addition, the binding free energies (ΔG_bind_) of W8 (−42.330 ± 3.341 kcal/mol) and BMS22 (−30.560 ± 6.076 kcal/mol) indicate that the high binding affinity is not necessarily accompanied by high inhibitory activity. Last but not least, the per-residue interaction analysis revealed that the contribution energy of ASP351 to binding was the most significant difference between BMS22 and W8, −2.195 kcal/mol and 1.707 kcal/mol, respectively. As a result, increasing the affinity between SMKIs and ASP351 of TGFβRImay effectively improve the inhibitory activity. The insights gained from this study could help with structure-guided optimization in searching for better SMKIs of TGFβRI.

## 1. Introduction

Transforming growth factor β (TGFβ) is one of the important members of the TGFβ superfamily [1]. Normally, TGFβ plays a crucial role in early embryonic development and tissue and organ growth, immunosuppression, tissue repair, and maintenance of tissue homeostasis [2,3,4,5,6,7]. However, TGFβs are frequently overexpressed in cancer, fibrosis, inflammation, or other disease states, and this excessive TGFβ production promotes the development of disease [8]. Interestingly, the TGFβ signal plays a dual role in tumorigenesis [9], exhibiting tumor suppressor properties during the early carcinogenesis and oncogenic properties during the late stage of malignancy [10,11]. In the latter, for example, the TGFβ signal stimulates tumor cell proliferation [12] and enhances stem cell properties while suppressing the sensitivity to anticancer drugs [13]. Additionally, the TGFβ signal interferes with the physiological functions of various immune cells, such as T cells and natural killer cells, and attenuates the inhibitory effect of immune cells on tumor cell migration [14,15,16]. Therefore, inhibiting the TGFβ signaling propagation is a key way for anticancer drug development. It is intensively researched in several clinical trials currently underway involving therapies targeting TGFβ signaling while other members of the TGFβ superfamily are under-represented in current trials [17]. Hence, here we focus on the TGFβ signaling pathway.

**Figure 1:**
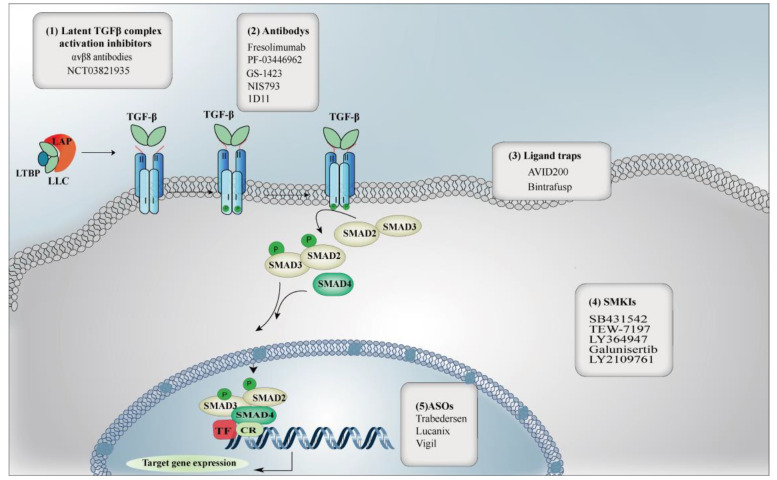
The representational blockers of the potential design targets along with TGF-β/Smad signaling [17,23]: (**1**) The latent TGFβ complex activation inhibitors: avβ8 antibodies and NCT03821935 [24]; (**2**) TGFβ monoclonal antibodies display outstanding specificity and extracellular mechanism: Fresolimumab, PF-03446962, GS-1423, NIS793, 1D11; (**3**) Ligand traps by fusing Fcγ to the extracellular domain of TGFβRII: AVID200 and Bintrafusp [25]; (**4**) Small-molecule kinase inhibitors (SMKIs) against TGFβRI/IIas ATP mimetics: SB431542, TEW-7197, LY364947, Galunisertib, LY2109761 [26]; (**5**) Antisense oligonucleotides and antisense RNA (ASOs) to downregulate ligands synthesis [27,28]: Trabedersen, Lucanix, Vigil.

In the TGFβ signaling pathway, an inactive TGFβ homodimer interacts with a latency-associated peptide (LAP) and a latent TGFβ-binding protein (LTBP) to form a larger latent complex (LLC) [18]. After the latent TGFβ matures, it is liberated from the latent complex and attaches to the TGFβ receptor type II (TGFβRII) and TGFβ receptor type I (TGFβRI) transmembrane serine/threonine kinase receptors. First, a TGFβ dimer binds to type II receptors, and then type I receptors are recruited to link with type II receptors to create an isomer, resulting in phosphorylation of TGFβRII. Next, Smad2/Smad3, the downstream substrate of TGFβRI, is activated to form trimeric complexes with Smad4 protein and translocates into the nucleus to regulate the expression of target genes (Figure 1) [19,20,21]. Thus, TGFβRI is the central node in the transmission of TGFβ signaling, and inhibiting the binding of Smad2/Smad3 can effectively block the TGFβ signaling pathway and reduce carcinogenic activity. SMKIs of TGFβRI are typically binding to the kinase domains as ATP mimics. It is worth noting that SMKIs tend to offer the benefits of being economical, productive, stable, and simple to deliver orally [22].

**Figure 2:**
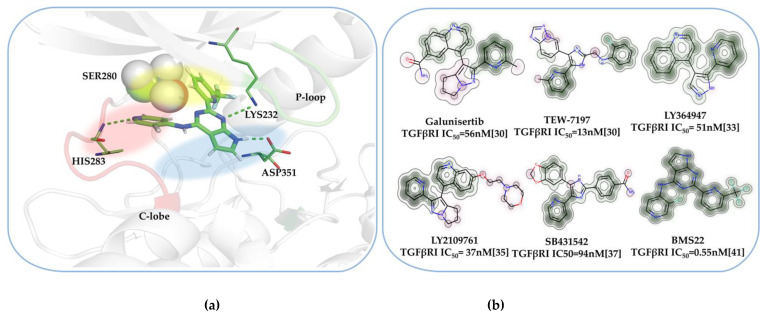
(**a**) A schematic view of the binding characteristics of BMS22 in the co-crystal structural domain depicting three colored regions: the hinge region (red), the selective hydrophobic pocket (yellow), and the solvent channel area (blue). As a typeIkinase inhibitor, BMS22 accommodates the DGF-in state of TGFβRI. (**b**) The similarity map of clinical trials testing TGFβRIinhibitors and BMS22 was predicted by Rdkit (http://rd.sourcceforge.net) based on MorganFingerprint, and the latter was the reference drug in this study (the greener the atom, the greater the similarity contribution). The source of the data literature is indicated in parentheses in Figure 2b.

There are several potentially active SMKIs of TGFβRI, such as Galunisertib [29,30], TEW-7197 [30,31], LY364947 [32,33], LY2109761 [34,35], and SB431542 [36,37] are undergoing clinical trials for treating cancers. Unfortunately, none of the inhibitors of TGFβRIhave been brought to market. It is necessary to further develop inhibitors for this target with potential druggability and high activity. Based on the findings of our previous studies [38,39,40], the pharmacophore model construction for the BMS22-TGFβRI complex (PDB ID: 6B8Y) [41], structure–activity relationship analysis, and scoring function evaluation, it is determined that: pyrrolotriazine TGFβRI inhibitors wedge with gatekeeper SER280 residue to weaken the stereo rejection in the pocket, and the inhibitors display a Y-shaped skeleton to coincide with the Y-type binding pocket (Figure 2a); the PMF and Ludi 1 are the top two functions for identifying the best ligand pose. In addition, the structural similarity map (Figure 2b) between BMS22 and the five clinical TGFβRI inhibitors reveals they share the Y-shaped molecular shape and some partially identical molecular fragments, such as a pyridine ring [42], and suggests that BMS22 brings together the active fragments of the five molecules in clinical studies, having clinical application potential and commercial worth. Further detailed BMS22 involves the majority of the marketed drugs’ fragments, such as pyrrolopyrimidine, pyridine and rifluoromethyl [42]. Based on the structure of this compound, it is promising to explore TGFβRI inhibitors with high druggability. Hence, BMS22 was chosen as an initial compound based on structure-based drug design to generate and optimize TGFβRI inhibitors. 

**Figure 3:**
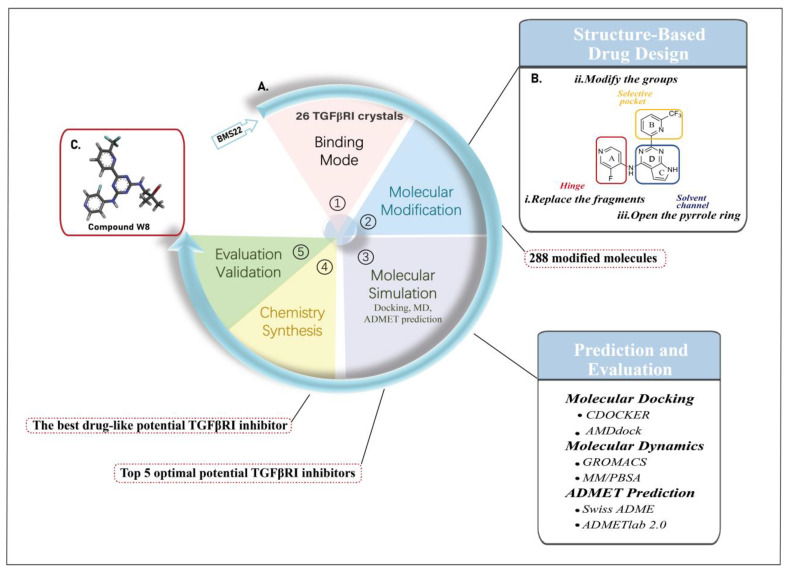
The flow chart to identify the optimal drug-like TGFβRIinhibitors ((**A**) the whole workflow; (**B**) a schematic of molecular modification; (**C**) the final screened molecule).

In this study, based on the analyses of the active sites on TGFβRI, we envisioned structural modifications of the pyrrolopyrimidine scaffold in BMS22 (Figure 3B), which resulted in three series of modified molecules: H-series (hinge region fragments modified), S-series (selective region fragments modified), and W-series (solvent channel fragments modified). Then, for screening out the optimal and potential druggable TGFβRIinhibitors, the previous compounds were subjected to molecular docking, ADMET prediction, and dynamics simulation. Meanwhile, the best molecule was synthesized and its inhibitory activity was evaluated. The work chart is shown in Figure 3. Our work could help to make tangible progress in the direction of the discovery of better drug-like SMKIs of TGFβRI.

## 2. Results and Discussion

### 2.1. The Binding Mode of the Co-Crystal of TGFβRI

Detailed interactions between protein and inhibitor as well as key residues in the protein-inhibitor binding interface could provide substantial information for the discovery and design of TGFβRIinhibitors. LigPlot^+^ is a program to show 2D representations of protein–ligand complexes from standard Protein Data Bank file input [43]. According to LigPlot^+^ interaction analysis (Table S1), the drug–target binding pattern of 26 distinct TGFβRIinhibitors displays the common features: (1) the hinge region HIS283 acts as a hydrogen bond donor to interact with nitrogen or oxygen in heterocyclic segments of ligands, which may be a key residue for inhibition; (2) the gatekeeper residue SER280 and hydrophobic residues such as ILE, LEU, and ALA establish a significant number of hydrophobic contacts with the ligand, contributing to increased ligand–acceptor affinity; (3) ASP351 forms a hydrogen bond with the ligand in the solvent channel and regulates the kinase stability as one of the characteristic residues of the DLG-motif (in the TGFβRIstructure, PHE has been mutated to LEU in the DFG motif). Additionally, LYS232 is also a high-frequency amino acid residue that forms a hydrogen bond. In the active pocket of 6B8Y (Figure 2a), the binding mechanism of BMS22 is consistent with the aforementioned general characteristics of TGFβRIinhibitors. On the one hand, BMS22 forms hydrogen bonds with three essential residues, HIS283, LYS232, and ASP351; on the other hand, BMS22 also generates hydrophobic contacts with ILE211, VAL219, ALA230, LEU278, ALA350, LEU340 in the selective active pocket. Furthermore, there is almost no substantial interaction between BMS22 and the P-loop in the solvent channel. In conclusion, improving the interactions with HIS283, LYS232, ASP351, SER280, and the P-loop region is critical for the design of TGFβRIinhibitors.

### 2.2. Results of Molecular Modification

Structure-based drug design is crucial in the development of several FDA-approved small-molecule kinase inhibitors, such as the Bcr-abl kinase inhibitor dasatinib [44]. As has been reported, most kinase inhibitors can form hydrogen bonds with the hinge region residues via nitrogen-containing heterocycles, which is a conserved mode of hydrogen bonding interaction for the SMKIs [45]. In response, the FDA-approved six-membered ring system as well as fused five- and six-membered ring systems, such as aminopyridines, aminopyrimidines, pyrrolopyridines, pyr-rolopyrimidines, purines, and imidazo triazine [23], were used to replace the hinge binder, pyridyl, in BMS22. Additionally, the volume of the gatekeeper residue determines ligand access to the active hydrophobic pocket in the kinase domain, which is closely related to kinase selectivity [46]. Our earlier research demonstrated that pyrrolotriazine inhibitors form a Y-shape binding with SER280, the gatekeeper residue in the ATP cleft of TGFβRI [39]. As a result, this Y-shape binding may improve the selectivity of TGFβRI inhibitors. Even though the pyrrole ring in BMS22 extends to the solvent channel, there is a weak connection between the pyrrole ring and the P-loop ring, therefore opening the pyrrole ring to branch chains for improving the utilization of the P-loop region. Figure 3B depicts the three modifications made to BMS22 based on the preceding analysis: i. substituting ring A with some hinge binders; ii. introducing the polar groups such as -SF_5_, -NH_2_, and -SONH_2_ to ring B to promote interaction with the SER280 as well as maintaining a Y-shape binding; iii. opening ring C and inserting flexible side chains such as -CH_2_CH_3_OH, -CH_2_CH_2_CH_2_CH_2_OH, -CH_2_C(CH_3_)_2_OH, or other five-membered tiny ring fragments, etc.

In accordance with the substantial understanding of BMS22’s binding mode and the confirmation of key residues in the active pocket, BMS22 was modified to produce 288 new molecules, including 124 H-series molecules, 19 S-series, and 145 W-series molecules (Figure S1), none of which were found to be entity molecules in the Scifinder database. All were continued to the next phase of molecular docking research.

### 2.3. Molecular Docking Analysis

**Figure 4:**
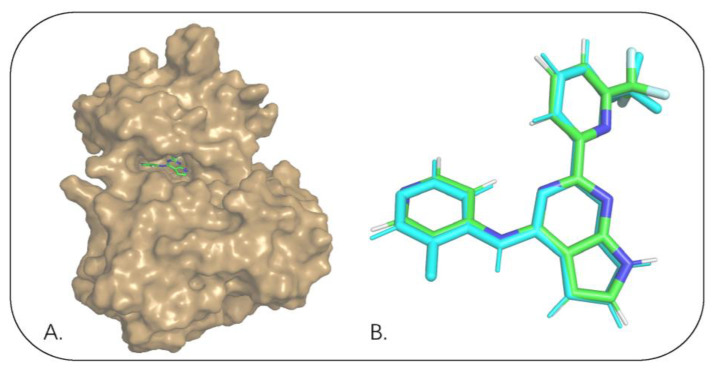
(**A**) The global diagram of the active pocket on the surface of TGFβRI(PDB ID: 6B8Y). (**B**) The re-docking validation: the crystal pose (Green) and re-docking pose (Cyans) of the ligand BMS22.

Molecular docking is used to analyze the conformation and orientation of small molecules into the active site of a bio-macromolecular target [47], which was conducted by the CDOCKER module [48] in this study. And all modified molecules were docked into the active site on TGFβRI (PDB ID:6B8Y) to obtain the docking score, binding affinity and estimated *K*_i_ value. Before this step, the docking pose of BMS22 and co-crystal conformation of 6B8Y were overlapped to validate the docking accuracy of the CDOCKER protocol. On the other hand, the ability of scoring functions in molecular docking to distinguish active and inactive molecules was evaluated in accordance with calculating the receiver operating characteristic curve (ROC). Then, the results show the root-mean-square deviation (RMSD) value between the docking pose and eutectic pose of BMS22 in the active pocket of 6B8Y was 0.2797 (Figure 4), which means the CDOCKER protocol can be applied effectively to obtain a reliable docking pose of TGFβRI inhibitors. Whereas, the AUC of the CDOCKER ENERGY is 0.539, which is an incredible indicator to distinguish the active pose of these docking ligands. Herein, the credible scoring function must be filtered out for this target. As shown in Table S2, the ideal scoring function for the TGFβRIdocking system to accurately obtain the most active conformation of the ligand is PMF with an AUC of 0.921 (>0.9 indicates great accuracy).

The results of molecular docking revealed that 30 modified compounds had excellent PMF values that were higher than 112.00 comparable to the reference molecule BMS22 (PMF = 113.32), including 11 molecules from the H-series, 11 molecules from the S-series, and 8 molecules from the W-series (Table S3–1 and Table S3–2). Except for H5 and S4, which formed only two hydrogen bonds with the active pocket, the other molecules formed three or four hydrogen bonds with key residues LYS232, HIS283, ASP351, SER280, or TYR249, as confirmed by manual review and had good hydrophobic interactions with non-polar amino acids such as ILE211, VAL219, LEU260, LEU278, and ALA350. This is consistent with most TGFβRIinhibitors in the crystal structure having the same mode of action. Table S3–1 summarized the PMF scores, binding affinity energies, estimated *K*_i_ values, and the key residues at the active site. The findings demonstrated that the compounds H1-H10 showed higher binding affinity and *K*_i_ values than BMS22. Meanwhile, H1 and H2, the top two PMF scoring molecules, may have potential TGFβRIinhibitory activity with substantially lower *K*_i_ values than the reference molecule (165.85 nM) of 4.87 nM and 8.50 nM, respectively. However, only S1 (−9.81 kcal/mol, 64.45 nM), S3 (−9.87 kcal/mol, 58.24 nM), S5 (−9.75 kcal/mol, 71.32 nM), and S6 (−9.68 kcal/mol, 80.26 nM) of the S-series showed a better binding affinity and *K*i than BMS22. Furthermore, the binding modes revealed that the introduction of the -SONH_2_ group may effectively enhance the hydrogen bonding of S1, S3, and S6 to SER280, which would potentially improve its selectivity for TGFβRI. For the W-series molecules, W1, W6, and W8 displayed strong columns of potential TGFβRI inhibition with predicted *K*_i_ values of 2.55 nM, 1.1 nM, and 2.16 nM, respectively. Subsequently, based on the above analysis, the molecules H1–H10, S1, S3, S5, S6, W1, W6, and W8 were subjected to ADMET property prediction to assess the drug-like properties.

### 2.4. ADMET Prediction

**Figure 5:**
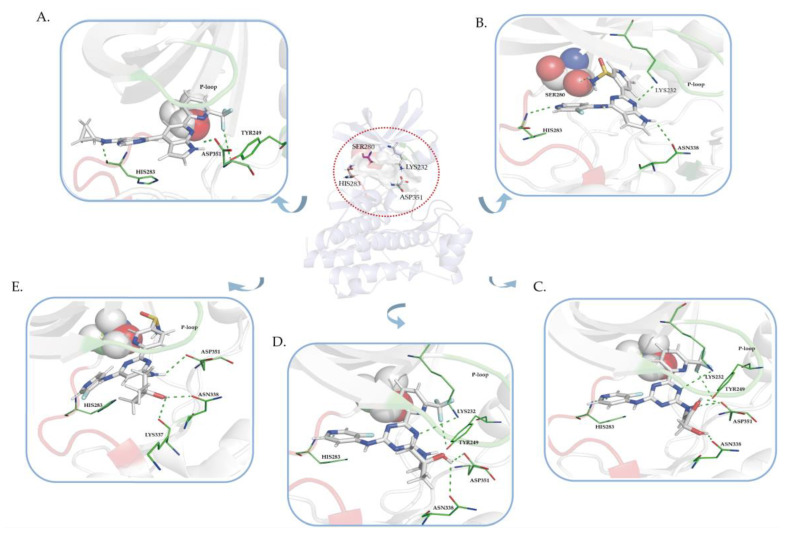
The input conformations of H1, S1, W1, W8 and S1W8 for MD simulation are displayed in figures (**A**–**E**) respectively.

In silico prediction of pharmacokinetic properties is an important component of pharmaceutical research and development and has been central to guiding hit-to-lead and lead-optimization efforts in the early phases of drug discovery [49]. In this research, the pharmacokinetic characteristics of modified compounds with high scoring, rational binding pose, and strongly predicted *K*_i_ were calculated by the SwissADME [50] and ADMETlab 2.0 web servers [51]. Table 1 shows the results of ADMET prediction for 17 selected molecules. All modified compounds were accepted by the Lipinski Rules and only H1, W1, W6, and W8 showed a negative value to P-gp substrates, while BMS22 did not, which indicated that they might degrade the antitumor resistance. What’s more, the introduction of cyclic fragments such as 1H-pyrrolo[3,2-c] pyridine (H3), pyrimidine-2 amine (H8), and other cyclic fragments could improve the predicted GI absorption, whereas the introduction of -SONH_2_ did not significantly increase GI absorption. Additionally, most modified molecules did not have the potential BBB permeability for minimal CNS side effects and low carcinogenicity, while only the S3 and W8 had low potential cardiotoxicity. Overall, ADMET predictions revealed that W8 had outstanding pharmacokinetic properties and high druggability as the most potential TGFβRIinhibitor, with a synthetic accessibility score of 3.10 (from 1(easy) to 10 (difficult)). Other research showed that the side chain, 1-amino-2-methyl-2-propanol, of W8, contained two common medicinal fragments of isopropyl alcohol and propan-1-amine, and 3-fluoropyridin-4-yl and 6-(trifluoromethyl)pyridin-2-yl also appeared frequently in drug molecules, so it was inferred that W8 had good potential medicinal properties [42]. Based on the molecular docking analysis, we retained H1, S1, W1, and the best drug-like molecule W8 to further estimate the target affinities by MD simulations. In addition, the (6-trifluoromethyl) pyridine in W8 was substituted with the pyridine-3-sulfinamide of molecule S1 to fuse the compound S1W8. With four hydrogen bonding contacts made with HIS283, ASP351, ASN337, and ASN338, S1W8 outperformed BMS22 in terms of a PMF score of 124.65, even though it had a binding affinity of −8.74 kcal/mol and *K*_i_ of 392.23 nM. The ADMET prediction showed that its Log*P* was lower than that of BMS22 and its other pharmacokinetic properties were equivalent to those of BMS22. In MD simulations, it was tested alongside H1, S1, W1, and W8. For the MD simulations, the input conformations of the aforementioned small molecules were listed in Figure 5.

### 2.5. MD Trajectories Analysis

In molecular biology and drug discovery, molecular dynamics (MD) simulations dramatically capture the behavior of proteins and other biomolecules in full atomic detail and at a very fine temporal resolution [52]. In this study, the best docking conformations of H1, S1, W1, W8, S1W8, and BMS22 were submitted to GROMACS 2020.3 software [53] to further assess the binding stability and figure out the key interactions between potential SMKIs and TGFβRIduring the dynamical progress. The root mean square deviation (RMSD), the root mean square fluctuation (RMSF), and hydrogen bond occupancy were also calculated on the basis of 100 ns trajectory for the best docking conformation of each system.

**Figure 6:**
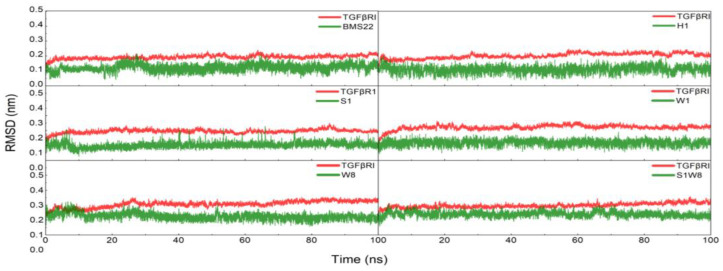
RMSDs of the TGFβRI backbone and ligands’ heavy atoms of six systems during MD simulations.

Firstly, the convergences of MD trajectories were evaluated by RMSDs of C_α_ atoms of the protein and the heavy atoms of ligands. As shown in Figure 6, the complex systems were stable as evidenced by the RMSD values of the TGFβRI and the five modified compounds, which fluctuated less below 0.30 nm. Furthermore, from the monitoring of the RMSD value of the heavy atoms of the ligands, we can justify roughly that the ligand can bind to the protein in a stable pose. Meanwhile, it has been seen that H1, S1, W1, W8, and S1W8 reached relative stabilities faster than the reference BMS22 system, with RMSDs convergent at about 0.10 ~ 0.20 nm. The trajectories were performed for conformational analysis and energy calculation.

**Figure 7:**
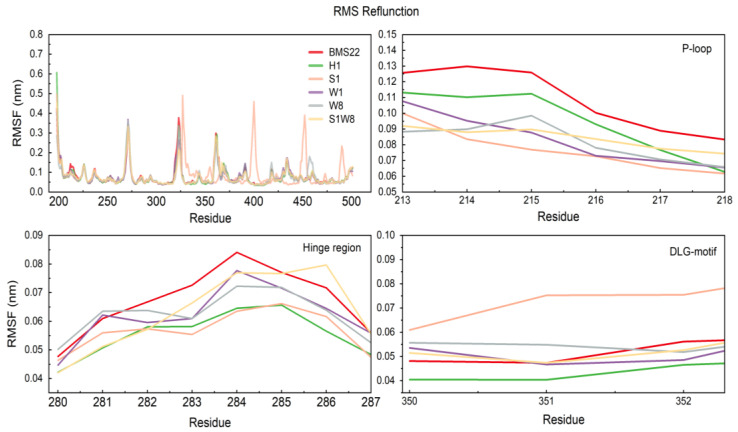
RMSF plot of the TGFβRI residues in different systems during the 100ns MD simulations.

The differences in the flexibility of the residues were analyzed by main-chain root mean square fluctuation (RMSF) versus the residue number of C_α_ based on the last 100 ns trajectory for each system, the result of which is given in Figure 7. For all six systems, it can be seen that the RMSF values of most residues have similar trends, and the RMSF values of the residues in active sites, such as P-loop, hinge region, and DLG-in motif, are all significantly more rigid than other regions. Moreover, the inhibition of all modified molecules in the P-loop region is stronger than that of BMS22, which not only enhances the stability of the P-loop, but also intensifies its contact with the active residues of the P-loop.

**Figure 8:**
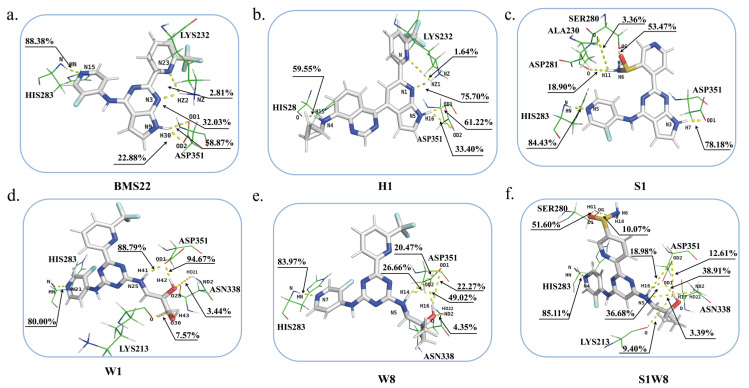
The 3D diagram of hydrogen bonds between TGFβRI and each potential inhibitor: (**a**–**f**) are BMS22, H1, S1, W1, W8 and S1W8, respectively.

The hydrogen bond is a critical nonbonding interaction in biological molecules’ spatial conformation and functions, thus the hydrogen bonds between the ligands and 6B8Y were monitored over the whole 100 ns MD simulation. Figure S3 shows the number of hydrogen bonds in each of the six TGFβRI complex systems during the simulation. It has been seen that the number of hydrogen bonds formed by molecules H1 and S1 with the active residues remained stable, while that in the TGFβRI-S1W8 system fluctuated greatly. Although S1W8 can generate up to six hydrogen bonds, the hydrogen interactions were less stable. The numbers of hydrogen bonds between the ligand and protein in W1 and W8 complexes were essentially stable in two or three. Stronger hydrogen bonds are indicated by a higher hydrogen bond occupancy. As shown in Table S4 and Figure 8, the occupancy rate of hydrogen bond between H1 and HIS283 was 59.55%, and the other compounds established at least one strong hydrogen bond in the 6B8Y hinge region, especially with the residue HIS283, which had a greater than 80% occupancy. This indicates that all molecules can bind effectively to hinge region residues, retaining the general properties of a potent kinase inhibitor; additional hydrogen bonding contacts may contribute to affinity due to the low solvent accessibility of the internal hydrophobic pocket in the 6B8Y active pocket. The -SONH_2_ group of S1 and S1W8 established the H-bond with the -OH of SER280, and the hydrogen occupancy rates were 53.47% and 10.07% respectively, both of which may increase the selectivity of TGFβRI. In this regard, enhancing the interactions with the gatekeeper residue SER280 in this area might improve the activity. W1 and W8 can even form more than two hydrogen bonds with ASP351. The top hydrogen bond occupancy between W1 and ASP351 was 94.67%, which was higher than that of BMS22 (58.87%), indicating that opening the pyrrole ring C and introducing the flexible side chains could enhance the hydrogen bonding interaction with ASP351. The largest hydrogen bond occupancy of the drug-like compound W8 with ASP351 was close to that of the reference molecule, which was 49.02%.

### 2.6. Binding Energy Analysis and Energy Decomposition

Although molecular docking is the gold standard in silico approach for identifying novel hit compounds that are active against the desired target receptor, there is still a need to improve the ability to distinguish true active ligands from inactive compounds [54]. In this context, using binding free energy evaluation approaches is a profitable strategy for revalidating docked ligand–protein complexes based on more reliable estimations of ligand–protein binding affinities than those obtained with simple scoring functions. To explore the differences in target affinity between each compound from the energy, the MM/PBSA calculation method was applied to calculate the binding free energies based on the last 20 ns equilibrium trajectories. The detailed information on binding free energies is listed in Table 2. Total binding free energies Δ*G*_bind_) were lower for W1, H1, W8, and S1W8 than for BMS22 (−30.560 ± 6.076 kcal/mol), barring the S1 (−28.466 ± 4.026 kcal/mol). In descending order, values of W1, H1, W8, and S1W8 were −46.992 ± 3.902 kcal/mol, −44.797 ± 4.962 kcal/mol, −42.330 ± 3.341 kcal/mol, −36.399 ± 5.052 kcal/mol, respectively. Besides, the energy component analysis revealed that the van der Waals energies and electrostatic interaction energy play a dominant role in the contribution to the total binding affinity of TGFβRI potential agents. Moreover, Δ*G*_vdw_ demonstrated a fairly high correlation toward Δ*G*_bind_ (Pearson’s r=0.614); although the value of Δ*G*_SA_ itself was small, the influence of Δ*G*_SA_ on the binding free energy could not be canceled (Pearson’s r = 0.681). Therefore, it has been indicated that increasing the van der Waals contacts with TGFβRI and the non-polar solvent interaction can substantially boost the binding affinity to the receptor to a certain extent.

**Figure 9:**
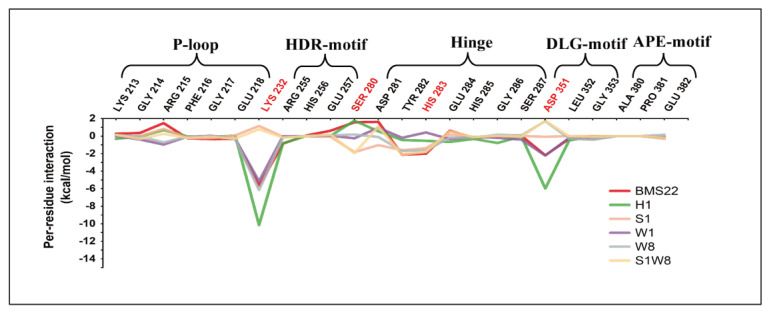
Contributions of free energy calculated by MM/PBSA method for key residues of TGFβRI.

Furthermore, it is critical to verify the interactions of modified molecules toward TGFβRI and to confirm the key residues in the binding interface. The results of binding free energy residue decomposition (Figure 9) showed that W1 and W8 could enhance the interaction with the P-loop region compared with the reference BMS22, and LYS232 in the active site contributed the most to the total binding energy. Obviously, H1 greatly increases the binding free energy with LYS232. Despite their weak interactions with LYS232, S1 and S1W8 both demonstrated good contributions to the total binding energy with the gatekeeper residue SER280 that were absent from the original complex. This implied that the addition of -SONH_2_ would improve the binding to the SER280, which might be advantageous for increasing the selectivity for TGFβRI. For the hinge-binding region residues, there was no significant difference in the contribution of binding energy in the hinge region. However, at DLG-motif, H1 improved the binding interaction of ASP351 significantly, and W1 bound to ASP351, similar to the original ligand BMS22, while the drug-like compound W8 displayed poor binding to ASP351. In the APE-motif region, there were no outstanding contributions or significant differences to the total binding for each molecule. Finally, because of its strong drug-like qualities, the compound W8 was further synthesized and kinase activity estimated.

### 2.7. Kinase Active Validation

ADP-Glo is a brand-new homogenous, bioluminescent assay for monitoring biological activities that produce ADP; as a result, it is ideally suited for determining enzyme activity when employing a variety of substrates [55]. It is a versatile test that can be utilized with ATPases, lipid kinases, sugar kinases, protein kinases, and many other types of kinases. In this study, the ADP-Glo technique was used to test the inhibition of the compound W8 on TGFβRIand its IC_50_ was evaluated to be 10.77 μM, indicating that it has a certain inhibitory impact on TGFβRIkinase and can be further examined as a lead chemical for TGFβRIinhibition.

## 3. Materials and Methods

### 3.1. Data Collection and Preparation

The architecture of the TGFβRI was initially discovered in 1999 [56]. To scrape the information on essential interactions of TGFβRI active pocket, 26 crystal structures (PDB ID: 1PY5, 1VJY, 1RW8, 3FAA, 3GXL, 3HMM, 2WOU, 2WOT, 3KCF, 2X7O, 3TZM, 4X2J, 4X2K, 4X2G, 4X0M, 4X2F, 5E8W, 5E8Z, 5FRI, 5USQ, 6B8Y, 5QIK, 5QIL, 5QIM, 5QU0, 5QTZ) were downloaded from the RCSB database (https://www.rcsb.org/) and analyzed by LigPlot^+^ 2.2.5 program(Hinxton, UK.) [43,57]. Before molecular docking, all the modified molecules were energy minimized by Discovery Studio 2020 (DS2020: https://www.3ds.com/), and TGFβRI(PDB ID: 6B8Y) was prepared and cleaned in the DS 2020 program to add the hydrogen atoms, complement the missing amino acid residues and optimize the side chain conformation.

### 3.2. Molecular Docking

The multiple poses generated by the professional search algorithm are ranked according to the scoring functions [58]. Therefore, the top docking pose for each ligand is scored by the optimal function. To confirm the accurate score function, the 50 active molecules were considered as the positive dataset and 300 inactive molecules of TGFβRI were identified as a negative dataset, which were randomly selected from the DUD-e database [59] (http://dude.docking.org) using the Find Diverse Molecules protocol of DS2020. All above compounds were docked into the ATP binding cleft of 6B8Y. Next, the twelve scoring functions, including CDOCKER ENERGY, CDOCKER INTERACTION ENERGY, PLP1, PLP2, PMF, PMF04, Jain, LigScore1, LigScore2, Ludi Energy Estimate 1 (Ludi 1), Ludi Energy Estimate 2 (Ludi 2), Ludi Energy Estimate 3 (Ludi 3), were used to evaluate the ligand binding pose in the active pocket. Then, the score functions were estimated by the area under the curve (AUC) of the receiver operating characteristic (ROC) curve using the Calculation ROC Curve tool of DS 2020 program. The AUC > 0.9 means excellent accuracy, 0.7–0.9 indicates moderate precision, while an AUC of 0.5 represents a random event [60].

The CDOCKER, a semi-flex docking method based on the full CHARMm forcefield in DS2020, was used to identify the optimal binding conformations of ligands and the common interactions between ligands and TGFβRI. Before docking, the binding site was edited according to the original ligand, BMS22, in 6B8Y by the “From Current Selection” protocol. Next, the “Top Hits” was set to “10”, “Pose Cluster Radius” was set to”0.5” and the other docking parameters were default. Finally, the optimal pose of each molecule with an excellent docking score was retained for the next analysis.

The estimated *K*_i_ is a useful value as it is more related to usually measured experimental parameters, as compared to the affinity. The estimated *K*_i_ and binding affinity of the designed inhibitors that were hit by CDOCKER were calculated using the Auto dock 4 protocol of AMDock 1.4 [61]. Initially, the "simple docking" option was selected to predict the binding mode of a single protein–ligand complex. Then, on the geometric center of the current ligand, a flexible box with a grid center (x = 5.3186, y = 8.8746, z = 5.0928) and optimum dimensions (x = 53, y = 53, y = 53) was defined using the "Center to hetero" option. The other parameters were set to their default values. Following docking, the conformation of each molecule that was consistent with the CDOCKER results was retained.

### 3.3. ADMET Prediction

The molecules with the high scoring, rational binding pose, and strong predicted *K*_i_ were evaluated by the SwissADME online server [50] and ADMETlab 2.0 [51], the pathway to access absorption, distribution, metabolism, excretion, and toxicity properties, such as water solubility (Log P), gastrointestinal absorption (GI), blood–brain barrier permeability (BBB), carcinogenicity, and hERG inhibition. Meanwhile, the rule-based filters from Lipinski’s rules were used to predict the drug-likeness, and the synthetic accessibility of molecules was measured on a scale of 1 to 10 [62]. On this basis, we preserved the compounds with the optimal drug-likeness for molecular dynamics study.

### 3.4. Molecular Dynamics Simulation

GROMACS 2020.3 software was utilized to evaluate the stability and the binding circumstance of a protein–ligand complex [53,63,64,65]. The initial structures and topologies for protein 6B8Y and its ligands were considered according to the results of molecular docking. Firstly, the topologies and parameters of each molecule were derived from SwissParam, a fast force field generation tool (https://www.swissparam.ch/) [66]. The protein 6B8Y and water were then parameterized by CHARMm36 force field [67] and TIP3P [68], respectively. The complex system was performed in an NVT ensemble at 300 K for 100 ps and subsequently in an NPT constant simulation at 1 bar for 100 ps. After both equilibration processes, all-atom systems were conducted for 100 ns MD simulation. The particle mesh Ewald method was used to evaluate the electrostatic interactions with a cut-off radius of 1.2 nm, and van der Waals interactions were switched between 1.0 and 1.2 nm. The H-bonds were constrained with LINCS, in the order of 4. The relative stable trajectories were used for further analysis, such as the root mean square distance (RMSD) analysis and per-residue root mean square (RMSF) analysis, and so on.

### 3.5. Binding Free Energy Calculation and Decomposition

Molecular mechanics/Poisson–Boltzmann surface area (MM/PBSA) is one of the precise approaches to evaluate binding free energies from the MD trajectories [69]. This computational theory is based on the following equations:

(1) $$\Delta G_{bind}=G_{complex}-\left(G_{reeptor}+G_{ligand}\right)$$

(2) $$\Delta G_{bind}=\Delta E_{MM}+\Delta G_{sol}-T\Delta S$$

(3) $$\Delta E_{MM}=\Delta E_{ini}+\Delta E_{vdw}+\Delta E_{ele}$$

(4) $$\Delta G_{sol}=G_{polar}+G_{apolar}$$

(5) $$\Delta G_{apolar}=\gamma \Delta SASA$$

Where Δ*G*_bind_ is the free energy of binding, Δ*E*_MM_ is the difference between the internal energies of molecules in a vacuum, and Δ*G*_sol_ is the difference between the free energies of solvation. Δ*S* represents the entropic change, while *T* represents the thermodynamic temperature. In addition, due to high computational costs and poor prediction accuracy, the conformational entropy change brought on by ligand binding (−*T*Δ*S* ) is canceled out. Δ*E*_MM_ can be subdivided into several terms, which is consistent with van der Waals energy (Δ*E*_vdw_), electrostatic interaction energy (Δ*E*_ele_) and (Δ*E_ini_*). However, the Δ*E_ini_*, such as a bond, angle, and torsion energy, is ignored usually. Moreover, Δ*G*_sol_ is the total of two energies: the polar energy (Δ*G*_polar_ also called Δ*G*_PB_) determined by the continuum solvent Poisson–Boltzmann model (PB) and the nonpolar energy (Δ*G*_apolar_ also called Δ*G*_SA_) determined by the change of the solvent accessible surface area (SASA). Therefore, Δ*G*_bind_ can be calculated using the following equation:

(6) $$\Delta G_{bind}=\Delta E_{vdw}+\Delta E_{ele}+\Delta G_{polar}+\Delta G_{apolar}$$

In this study, the free energy of binding was evaluated by exacting the last 20 ns trajectory from the whole MD process, using the g_mmpbsa tool [70] according to the above formulas. Furthermore, the per residual average binding free energy contribution was also quantified by the Δ*E*_vdw_, Δ*E*_ele_, Δ*G*_polar_ and Δ*G*_apolar_ which were used to differentiate crucial residues in the protein–ligand interaction.

### 3.6. Experimental Sections

#### 3.6.1. General Procedure of Synthetic Reaction

##### General Information

All evaporations were carried out in a vacuum with a rotary evaporator. Analytical samples were dried under a vacuum (1–5 mmHg) at room temperature. Thin layer chromatography (TLC) was performed on silica gel plates, and spots were visualized by UV light (214 and 254 nm). Purification by column and flash chromatography was carried out using silica gel (200–300 mesh). Solvent systems were reported as mixtures by volume. All NMR spectra were recorded on a Bruker 400 (400 MHz) spectrometer. ^1^H chemical shifts were reported as δ values in ppm with the deuterated solvent as the internal standard. Data are reported as follows: chemical shift, multiplicity (s = singlet, d = doublet, t = triplet, q = quartet, br = broad, m = multiplet), coupling constant (Hz), and integration. LCMS spectra were obtained on an Agilent 1200 series 6110 or 6120 mass spectrometer with electrospray ionization and excepted as otherwise indicated, the general LCMS condition was as follows: Waters X Bridge C18 column (50 mm × 4.6 mm ×3.5 um), Flow Rate: 2.0 mL/min, the column temperature: 40 °C.

**Scheme 1:**
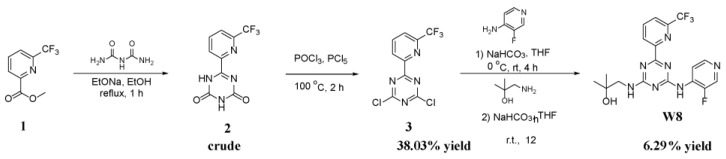
Synthetic route for the compound W8.

The compound W8 was synthesized based on a previously described synthesis method [71] as depicted in Scheme 1. The NMR spectra and LCMS spectra of synthesized compounds were shown in Figure S4–S6 and Report 1–3 in the Supporting File. 

##### The Synthesis of 6-(6-(Trifluoromethyl)Pyridin-2-yl)-1,3,5-Triazine-2,4(1H,3H)-Dione (2 in Scheme 1)

To a solution of freshly prepared NaOEt from Na (384 mg, 16.70 mmol) in ethanol (50 mL) was added methyl 6-trifluoromethylpicolinate (3.30 g, 16.09 mmol) and biuret (848 mg, 8.22 mmol). The resulting mixture was stirred to reflux for 1 h and then concentrated. The residue was poured into water and treated with saturated aqueous NaHCO_3_ to adjust the pH to 7.0. The precipitated solid was collected by filtration and dried under a vacuum to give the desired compound 2 (2.39 g, crude) as a white solid. ^1^H-NMR (400 MHz, DMSO-d_6_) δ 9.67 (br, 1H), 8.38 (d, J = 8.0 Hz, 1H), 8.14 (t, *J* = 7.6 Hz, 1H), 7.93 (d, *J* = 7.6 Hz, 1H). LC-MS: m/z for C_9_H_6_F_3_N_4_O_2_ (M+H)^+^: calculated 259.04; found 259.1.

##### The Synthesis of 2,4-Dichloro-6-(6-(Trifluoromethyl)Pyridin-2-yl)-1,3,5-Triazine (3 in Scheme 1)

To a solution of 6-(6-trifluoromethyl-pyridine-2-yl)-1,3,5-triazine-2,4(1H,3H)-dione (2.05 g, 7.94 mmol) in POCl_3_ (29 mL) was added PCl_5_ (13.80 g, 66.27 mmol). The mixture was stirred at 100 ℃ for 2 h and then concentrated. The residue was dissolved in EtOAc and then washed with saturated aqueous NaHCO_3_. The organic layer was separated, dried over anhydrous Na_2_SO_4_, and then concentrated under reduced pressure. The residue was purified by flash chromatography (silica gel, petroleum ether: EtOAc = 5:1) to give the desired product 3 (900 mg, 38.03% yield) as a white solid. ^1^H-NMR (400 MHz, CDCl_3_) δ 8.75 (d, *J*=7.6 Hz, 1H), 8.16 (t, *J* = 8.0 Hz, 1H), 7.96 (dd, *J* = 8.0, 0.8 Hz, 1H). LC-MS: m/z for C_9_H_4_C_l2_F_3_N_4_(M+H)^+^: calculated 294.98; found 295.1.

##### The Synthesis of 1-((4-((3-Fluoropyridin-4-yl)Amino)-6-(6-(Trifluoromethyl)Pyridin-2-yl)-1,3,5-Triazin-2-yl)Amino)-2-Methylpropan-2-ol (W8 in Scheme 1)

To a mixture of 2,4-dichloro-6-(6-(trifluoromethyl)pyridin-2-yl)-1,3,5-triazine (88 mg, 0.30 mmol) and NaHCO_3_ (50 mg, 0.60 mmol) in dry THF (10 mL) was added 3-fluoropyridin-4-amine (34 mg, 0.30 mmol). The mixture was stirred at 0 ℃ for 4 h. Then, 1-amino-2-methylpropan-2-ol (27 mg, 0.30 mmol) was added to the above mixture. The mixture was stirred at room temperature for 12 h. The reaction mixture was diluted with EtOAc (30 mL) and water (10 mL). The organic layer was washed with brine (10 mL), dried over anhydrous Na_2_SO_4_, and then concentrated under reduced pressure. The residue resulting from this step was purified by Prep-HPLC to give the desired product W8 (8 mg, 6.29% yield) as a white solid. ^1^H-NMR (400 MHz, DMSO-*d*_6_, 333 K) δ 9.55 (br, 1H), 8.53–8.57 (m, 1H), 8.46 (s, 1H), 8.24–8.43 (m, 3H), 8.03 (d, *J* = 7.6 Hz, 1H), 7.50–7.59 (m, 1H) 4.32–4.44 (m, 1H), 3.36–3.45 (m, 2H), 1.14 (s, 6H). LC-MS: m/z for C_18_H_18_F_4_N_7_O (M+H)^+^: calculated 424.15; found 424.2.

#### 3.6.2. Kinase Active Validation

The ADP-Glo kinase assay was adopted to evaluate the inhibition effect of W8 toward TGFβRI in a 1× kinase buffer (50 mM Tris pH 7.5, 10 mM MgCl_2_, 200 nM NaCl, 0.1% BSA, 1mM DTT) at room temperature. The compound was dissolved in 100% DMSO to prepare the working solution at a concentration of 1.0 mM. The ADP-Glo kinase assays were conducted in wells of a 384-well white plate in the 20 μL final solution. Firstly, the 50 nL compound solution and 2.5 μL 2x kinase solution (TGFβRI) were added in the compound wells while the positive wells only added 2.5 μL 2x kinase solution (TGFβRI). Then, the negative control wells added the 2.5 μL 1x kinase buffer. Next the 2.5 μL 2x substrate (ATP) was added to each well and the reactions were incubated for 120 min. Furthermore, the 5 μL of ADP-Glo R1 agent was added for 180 min reaction time. Subsequently, the 10 μL of ADP-Glo R2 agent was added to incubate for 60 min. All experiments were carried out following the manufacturer’s instructions (Promega). The data were collected in Envision and the IC_50_ values were calculated in Graph Pad Prism 5.

## 4. Conclusions

The highly active SMKIs of TGFβRIhave been involved in various cancer treatments, including non-small cell lung carcinoma, hepatocellular carcinoma, glioma, pancreatic cancer, and scirrhous gastric cancer targeted therapies, and so on. However, no TGFβRI-listed medicine has been effectively created today. In this study, the structural domain of TGFβRI kinase was divided into the hinge region, hydrophobic selective region, and solvent channel region to guide the modification conducted on BMS22 based on the pyrrolo-pyrimidine backbone. The critical residues of TGFβRI were also identified. Then, the preferential binding conformations, drug-like properties, and the target affinity of the modified compounds were evaluated by computational simulations such as molecular docking, ADMET predictions, and molecular dynamics simulations. As a result, four potential TGFβRI inhibitors W1, H1, W8, and S1W8 having better binding free energies than that of BMS22 were obtained. Among these molecules, the ADMET prediction of W8 exhibited high water solubility, good GI absorption, and sufficient drug safety (non-carcinogenic, non-teratogenic, and non-cardiotoxic). Further experiments proved that the target’s half maximal inhibitory concentration value against TGFβRI was about 10 μM, which was somewhat less inhibitory than that of the reference molecule BMS22 but has an exceptional drug likeness and may be applied clinically. It defies the Δ*G*_bind_ prediction and illustrates that high binding energy does not necessarily result in a substantial inhibitory action. Meanwhile, the binding free energy on the per-residue level showed that the contribution of ASP351 in the DLG-motif of W8 changed from −2.194 kcal/mol to 1.706 kcal/mol compared with the BMS22, which may prove that ASP351 has a vital influence in the active site of TGFβRI.

Therefore, we can conclude that the high binding affinity may not necessarily lead to high inhibitory activity, and that interaction with certain amino acids, such as ASP351, is the crucial factor of activity. The subsequent modification of the TGFβRI inhibitor should focus on improving the interaction with ASP351, which is what we will study in the next stage. All the above may give structure-guided optimization in searching for the better SMKIs of TGFβRI. 

## Figures and Tables

**Table 1:** ADMET properties of the designed molecules.

Compound ID	Medicinal Chemistry	Physicochemical Property		Absorption	Distribution	Metabolism	Toxicity		Synthetic Accessibility ^b^
	Lipinski Rules	Log*P* ^a^	P-gp Substrate	GI Absorption	BBB Penetration	CYP2D6 Inhibitor	Carcinogenicity	hERG Blockers	
H1	Accepted	4.434	NO	LOW	YES	YES	NO	YES	3.10
H2	Accepted	4.473	YES	LOW	NO	YES	NO	YES	3.06
H3	Accepted	4.003	YES	HIGH	NO	YES	YES	YES	2.82
H4	Accepted	4.153	YES	HIGH	NO	YES	NO	YES	2.75
H5	Accepted	0.834	YES	HIGH	NO	YES	NO	YES	2.98
H6	Accepted	4.508	YES	LOW	NO	YES	NO	YES	3.11
H7	Accepted	3.852	YES	HIGH	NO	YES	NO	YES	3.05
H8	Accepted	0.493	YES	HIGH	NO	YES	NO	YES	2.91
H9	Accepted	4.203	YES	HIGH	NO	YES	NO	YES	2.93
H10	Accepted	2.344	YES	LOW	NO	YES	NO	YES	3.02
S1	Accepted	0.944	YES	LOW	NO	NO	NO	YES	3.49
S3	Accepted	1.720	YES	LOW	NO	NO	YES	NO	3.51
S5	Accepted	1.080	YES	LOW	NO	NO	NO	YES	3.50
S6	Accepted	1.630	YES	LOW	NO	NO	NO	YES	2.56
W1	Accepted	1.358	NO	LOW	NO	NO	NO	YES	3.22
W6	Accepted	3.430	NO	LOW	NO	NO	NO	YES	4.29
W8	Accepted	2.386	NO	HIGH	NO	NO	NO	NO	3.10
S1W8	Accepted	0.381	YES	LOW	NO	YES	NO	NO	3.87
BMS22	Accepted	4.434	YES	HIGH	NO	YES	NO	NO	3.10

^a^ Log of the octanol/water partition coefficient. Optimal: 0~3; ^b^ Synthetic accessibility score: from 1 (very easy) to 10 (very difficult).

**Table 2:** Binding free energies and corresponding energy components between the TGFβRI pocket and agents by MM/PBSA during the last 20ns MD simulations(kcal/mol).

Compound ID	Δ*E*_vdw_^a^	Δ*E*_ele_^b^	Δ*G*_MM_^c^	Δ*G*_PB_^d^	Δ*G*_SA_^e^	Δ*G*_bind_^f^
W1	−50.57 9 ± 3.021	−43.260 ± 5.632	−93.933 ± 5.471	52.089 ± 3.315	−5.242 ± 0.203	−46.992 ± 3.902
H1	−57.374 ± 3.601	−60.696 ± 5.444	−118.071 ± 8.208	78.628 ± 3.503	−5.353 ± 0.201	−44.797 ± 4.962
W8	−53.096 ± 2.804	−28.215 ± 1.848	−81.310 ± 3.683	44.303 ± 2.155	−5.321 ± 0.195	−42.330 ± 3.341
S1W8	−54.209 ± 3.598	−32.781 ± 8.034	−86.990 ± 6.865	56.018 ± 4.121	−5.427 ± 0.204	−36.399 ± 5.052
BMS22	−44.667 ± 3.130	−47.651 ± 9.194	−92.318 ± 8.797	66.294 ± 4.468	−4.536 ± 0.185	−30.560 ± 6.076
S1	−43.853 ± 2.726	−29.833 ± 4.672	−73.686 ± 4.405	49.486 ± 2.773	−4.265 ±0.189	−28.466 ± 4.026
Pearson’s r ^g^	0.614	0.395	0.582	−0.119	0.681	1

^a^ van der Waals interaction. ^b^ electrostatic interaction. ^c^ gas-phase interaction energy; ^d^ polar contribution of solvation effect. ^e^ nonpolar contribution of the solvation effect. ^f^ binding free energy. ^g^ the correlation coefficient of each energy component with the total binding energy.

## Data Availability

Data is contained within the article and Supplementary Materials.

## Supplementary Materials

The following supporting information can be downloaded at: https://www.mdpi.com/1424-8247/15/10/1264/s1, Table S1: The key amino acid residues interacting with ligands in the crystal structure; Table S2: The results of AUCs of scoring functions; Table S3–1: The information of the designed molecules is supported by docking to the receptor. (PDB ID:6B8Y); Table S3–2: The chemical structures of 30 modified molecules; Table S4: Occupancy rates of hydrogen bonds between TGFβRI and each potential inhibitor; Figure S1: The chemical structures of modified molecules from hinge region (in red), solvent channel region(in blue), and selective pocket region (in yellow); Figure S2: 3D interaction pattern diagram between the protein and modified molecules in 6B8Y; Figure S3: Number of hydrogen bonds of five designed molecules and reference ligand BMS22 combined with TGFβRI during 100 ns MD simulations; Figure S4: ^1^H-NMR spectrum of 6-(6-(trifluoromethyl)pyridin-2-yl)-1,3,5-triazine-2,4(1H,3H)-dione; Figure S5: ^1^H-NMR spectrum of 2,4-dichloro-6-(6-(trifluoromethyl)pyridin-2-yl)-1,3,5-triazine; Figure S6: ^1^H-NMR spectrum of 1-((4-((3-fluoropyridin-4-yl)amino)-6-(6-(trifluoromethyl)pyridin-2-yl)-1,3,5-triazin-2-yl)amino)-2-methylpropan-2-ol; LC-MS report 1: 6-(6-(trifluoromethyl)pyridin-2-yl)-1,3,5-triazine-2,4(1H,3H)-dione; LC-MS report 2: 2,4-dichloro-6-(6-(trifluoromethyl)pyridin-2-yl)-1,3,5-triazine; LC-MS report 3: 1-((4-((3-fluoropyridin-4-yl)amino)-6-(6-(trifluoromethyl)pyridin-2-yl)-1,3,5-triazin-2-yl)amino)-2-methylpropan-2-ol.
