# Supplementary material for: Structure-Guide Design and Optimization of Potential Druglikeness Inhibitors for TGFβRI with the Pyrrolopyrimidine Scaffold
Source: Pharmaceuticals (Basel). 2022 Oct 13;15(10):1264. doi: 10.3390/ph15101264 (PMC9653795; doi:10.3390/ph15101264)

Supplementary data

for

Structure-Guide Design and Optimization of Potential Druglikeness Inhibitors for TGFβRIwith the Pyrrolopyrimidine Scaffold

Dan Meng [1, 2, 3], Jiali Xie [1, 2,3], Yihao Li [1], Ruoyu Li [1,2,3], Hui Zhou [1,2,3,] and Ping Deng [1,2,3,]**

[1] College of Pharmacy, Chongqing Medical University, Chongqing, 400016, China.

[2] Chongqing Research Center for Pharmaceutical Engineering, Chongqing, 400016, China.

[3] Chongqing Key Research Laboratory for Quality Evaluation and Safety Research of APIs, Chongqing, 400016, China.

* Correspondence: hzhou@cqmu.edu.cn; 100865@cqmu.edu.cn

Table S1 The key amino acid residues interacting with ligands in the crystal structure

PDB ID	Active data	No.of H-bonds	Hydrogen Bonds	Hydrophobic	NO.of Hydrophobic interaction
1PY5	IC_{50}=104 nM	2	**HIS283**, **ASP351**	ILE211,VAL219,ALA230,VAL231,LYS232,LEU260,LEU278,LEU340,**SER280**,ASP281,TYR 282,GLY286	12
1VJY	IC_{50}=4 nM	3	LYS232, **HIS283**, **ASP351**	ILE211,VAL219,ALA230,TYR249, LEU260, **SER280**,ASP281,TYR282, ,GLY286 LEU340,LEU278	11
1RW8	IC_{50}=175 nM	1	**HIS283**	ILE211,LYS213,VAL219,ALA230,LYS232,TYR249,LEU260,LEU278,**SER280**,ASP281,LYS3 37,ASN338,LEU340,ASP351	14
3FAA	Ki=7.1 nM	3	**HIS283**, **ASP351**, **LYS232**	ILE211,ALA230,GLU245,TYR249,LEU260,LEU278,S**ER280**,ASP281,TYR282,ASN338,LE U340	11
3GXL	IC_{50}=25 nM	3	**LYS32**, **HIS83** (double H-bonds)	ILE11,GLY14,VAL19,ALA30,GLU45,TYR49,LEU78,**SER80**,ASP81,TYR82,GLY86,LYS137, LEU140, **ASP151**	14
3HMM	IC_{50}=25 nM	2	**LYS32**, **HIS83**	VAL19,ALA30,GLU45,TYR49,LEU60,LEU78,VAL79,TYR82,**SER80**,ASP81,LYS137,ASN1 38,LEU140, **ASP151**	14

2WOU	IC_{50}=72 nM	2	**HIS283**, ASP290	ILE211,VAL219,ALA230,**LYS232**,LEU260,LEU278,**SER280**,ASP281,TYR282,GLY286,LEU340,**ASP351**	12
2WOT	IC_{50}=44 nM	2	**HIS283**, **LYS232**	ILE211,VAL219,ALA230,LEU260,LEU278,VAL279,**SER280**,ASP281,TYR282,GLU284,GLY286,ASN338,LEU340,ALA350,**ASP351**	15
3KCF	Ki=35 nM	2	**LYS232**, **HIS283**	ILE211,ALA230,GLU245,TYR249,LEU260,PHE262,LEU278,VAL279,**SER280**,ASP281,TYR282,LYS337,ASN338,LEU340,**ASP351**	15
2X7O	IC_{50}=34 nM	2	ASP281, **HIS283**	ILE211,GLY212,VAL219,ALA230,LYS232,GLU245,LEU260,LEU278,**SER280**,TYR282,GLU284,HIS285,GLY286,APS290,ARG294,LEU340	16
3TZM	NR	2	**HIS283**, **LYS232**	ILE211,GLY214,ARG215,ALA230,LEU260,LEU278,**SER280**,ASP281,TYR282,LYS335,LYS337,ASN338,LEU340,**ASP351**	14
4X2J	NR	4	**HIS283**, TYR249, **ASP351**, GLU245	ILE211,VAL219,ALA230,**LYS232**,LEU260,**SER280**,ASP281,TYR282,GLY286,LEU340	10
4X2K	NR	3	**HIS283**, ASP281, ASP290	ILE211,VAL219,ALA230,**LYS232**,,LEU260,**SER280**,ASP281,TYR282,GLY286,LEU340	10
4X2G	NR	3	ASP281, **HIS283**, ASP290	ILE211,VAL219,ALA230,LEU260,**SER280**,TYR282,SER287,LEU340,TYR603	9
4X0M	NR	1	ASP281	ILE211,VAL219,ALA230,LEU260,TYR282,**HIS283**,LEU340	7
4X2F	NR	3	**HIS283**, ASP281, ASP290	ILE211,ALA230,,LEU260,TYR282,SER287,LEU340	6
5E8W	NR	3	ASP281, LYS337, **HIS283**	ILE211,GLY212,LYS213,GLY214,VAL219,ALA230,LYS232,LEU260,**SER280**,TYR282,GLY286,LEU340,**ASP351**	13
5E8Z	NR	3	**LYS232**, ASP281, **HIS283**	ILE211,LYS213,GLY214,ALA230,LEU260,TYR282,GLU284,GLY286,SER287,ASP290,LYS337,LEU340,**ASP351**	13
5FRI	IC_{50}= 1.9 nM	2	**HIS283**(double H-bonds)	ILE211,VAL219,ALA230,**LYS232,**LEU260,**SER280**,ASP281,TYR282,GLU284,GKY286,LEU340,ALA350,**ASP351**	13
5USQ	NR	2	**HIS283**, ASP290	ILE211,ALA230,**LYS232**,LEU278,VAL279,**SER280**,ASP281,TYR282,GLY286,SER287,LEU340,**ASP351**	12
6B8Y	IC_{50}=0.55 nM	3	**HIS283**, **ASP351**, **LYS232**	VAL219,ALA230,LEU260,LEU278,VAL279,**SER280**,ASP281,TYR282,LYS337,ASN338,LEU340,	11
5QIK	IC_{50}=24 nM	3	**LYS232**, **ASP351**, **HIS283**	ILE211,LYS213,GLY214,ALA230,LEU260,**SER280**,ASP281,TYR282,GLU284,GLY286,LYS337,LEU340	12

5QIL	IC_{50}=3 nM	2	**HIS283**, **LYS232**	ILE211,LYS213,GLY214,AL219,ALA230,TYR249,LEU260,LEU278,**SER280**,ASP281,GLY286,LEU340, **ASP351**	13
5QIM	IC_{50}=6 nM	2	**HIS283**, **LYS232**	ILE211,GLY214,VAL219,ALA232,GLU245,LEU260,PHE262,LEU278,VAL279,**SER280**,ASP281,TYR282,GLU284,GLY286,LEU340	15
5QU0	IC_{50}=1.6 nM	2	**LYS232**, **HIS283**	ILE211,VAL219,ALA230,LEU278,**SER280**,ASP281,TYR282,LEU340,**ASP351**	9
5QTZ	IC_{50}=10 nM	2	**HIS283**, **LYS232**	ILE211,VAL219,ALA230,TYR249,LEU260,LEU278,**SER280**,ASP281,TYR282,LYS337,LEU340,**ASP351**	12

Table S2. The results of AUCs of scoring functions.

Score Property	Area under ROC Curve	ROC Evaluation	The ROC were calculated by DS 2020 program.
CDOCKER_ENERGY	0.539	Fall	
CDOCKER_INTERACTION_ENERGY	0.751	Fair	
PLP1	0.832	Good	
PLP2	0.828	Good	
PMF	0.921	Excellent	
PMF04	0.864	Good	
Jain	0.761	Fair	
LigScore1_Dreiding	0.745	Fair	
LigScore2_Dreiding	0.821	Good	
Ludi_1	0.871	Good	
Ludi_2	0.859	Good	
Ludi_3	0.878	Good	

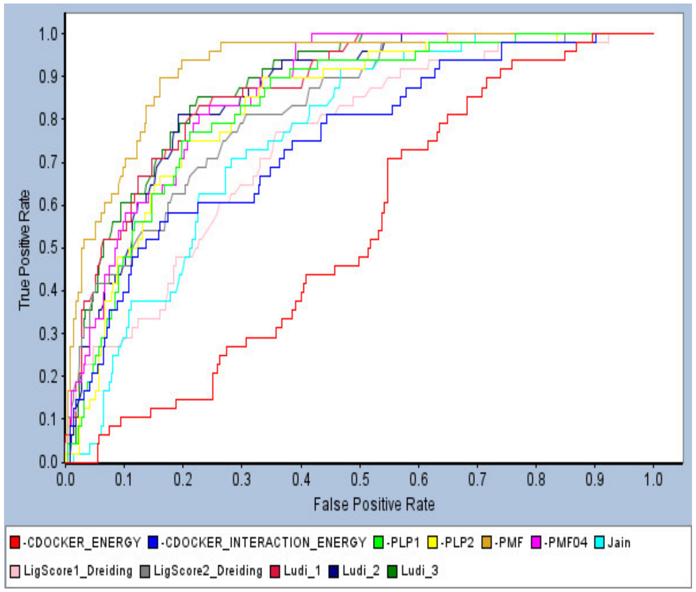

Table S3-1. The information of the designed molecules is supported by docking to the receptor. (PDB ID:6B8Y).

Compound ID	PMF	Affinity (kcal/mol)	K_i (nm)	H-Bond	Hydrophobic interaction
H1	127.36	-11.34	4.87	TYR249,HIS283,ASP351	ILE211,VAL219,ALA230,LEU278,TYR282,LEU340, ALA350
H2	124.63	-11.01	8.50	TYR249,HIS283,ASP351	ILE211,VAL219,ALA230,LYS232,LEU278,TYR282,LEU340,ALA350
H3	123.1	-9.27	160.34	LYS232,TYR249,HIS283,ASP351	ILE211,VAL219,ALA230,LEU278,LEU340,ALA350
H4	120.37	-9.47	110.61	LYS232,TYR249,HIS283,ASP351	ILE211,VAL219,ALA230,LEU260,LEU278,LEU340, ALA350
H5	119.48	-9.84	61.27	LYS232,HIS283	ILE211,VAL219,ALA230,TYR249,LEU260,LEU278, LEU340,ALA350
H6	118.83	-9.43	122.4	TYR249,HIS283,LYS232	ILE211,VAL219,ALA230,LEU278,LEU340,ALA350
H7	115.74	-9.37	135.44	LYS232,TYR249,HIS283,ASP351	ILE211,VAL219,ALA230,LEU260,LEU278,TYR282,LEU340,ALA350
H8	114.31	-9.5	108.76	LYS232,HIS283,ASP281,ASP351	ILE211,VAL219,ALA230,TYR249,LEU260,LEU278, LEU340,ALA350
H9	113.87	-9.87	61.27	LYS232,TYR249,HIS283,	ILE211,VAL219,ALA230,LEU278,LEU340,ALA350
H10	113.39	-9.39	130.95	LYS232,TYR249,HIS283,GLU284, ASP351	ILE211,VAL219,ALA230,LEU260,LEU278,LEU340, ALA350
H11	112.63	-8.92	289.47	LYS232,HIS283,ASP351	ILE211,VAL219,ALA230,TYR249,LEU260,LEU278,LEU340, ALA350
S1	125.89	-9.81	64.45	LYS232,SER280,HIS283,ASN338	ILE211,VAL219,ALA230,LEU260,LEU278,LEU340, ALA350
S2	124.07	-8.63	472.25	ALA230,LYS232,SER280,HIS283,ASP351,ASN338	VAL219,LEU260,LEU340,ALA350
S3	123.01	-9.87	58.24	LYS232,SER280,HIS283,ASP351	ILE211,VAL219,ALA230,LEU260,LEU278,LEU340, ALA350
S4	122.36	-8.46	608.31	SER280,HIS283	ILE211,VAL219,ALA230,LEU260,LEU278,LEU340, ALA350
S5	121.53	-9.75	71.32	LYS232,TYR249,HIS283,ASP351	ILE211,VAL219,ALA230,LEU340,ALA350
S6	121.22	-9.68	80.26	LYS232,SER280,HIS283,ASP351	ILE211,VAL219,ALA230,LEU260,LEU278,LEU340, ALA350
S7	121.21	-8.28	856.56	ALA230,LYS232,TYR249, LEU278,HIS283,ASP351	ILE211,VAL219,LEU260,LEU340,ALA350
S8	120.98	-8.31	810.47	LYS232,HIS283,ASP351	ILE211,VAL219,ALA230,LEU260,LEU278,LEU340, ALA350
S9	120.8	-8.65	456.58	LYS232,TYR249,HIS283,ASP351	ILE211,VAL219,ALA230,LEU260,LEU278,LEU340, ALA350
S10	119.36	-8.91	293.4	LYS232,LEU278,HIS283,ASP351	ILE211,VAL219,ALA230,LEU260,LEU340,ALA350
S11	112.25	-8.72	405.70	LYS232, HIS283,ASP351	ILE211,VAL219,ALA230,LEU260,LEU278,LEU340, ALA350
W1	123.06	-7.63	2.55	LYS232,TYR249,HIS283,ASP351,ASN338	ILE211,VAL219,ALA230,LEU260,LEU278,ALA350, LEU340
W2	116.8	-8.87	314.96	LYS232,TYR249,HIS283,ASP351,ASN338	ILE211,VAL219,ALA230,LEU260,LEU340
W3	116.42	-8.7	419.62	LYS232,TYR249,HIS283,ASP351	ILE211,VAL219,ALA230,LEU260,LEU340
W4	115.12	-8.68	434.03	LYS232,TYR 249,HIS283,LYS337,ASP351	ILE211,VAL219,ALA230,LEU260,LEU340
W5	114.08	-8.71	412.61	LYS232,TYR249,HIS283,ASP351	ILE211,VAL219,ALA230,LEU260,LEU340
W6	113.36	-8.13	1.1	LYS232,TYR249,HIS283,ASP351,ASN338	ILE211,VAL219,ALA230,LEU260,LEU278,LEU340, ALA350
W7	112.99	-8.65	456.58	LYS232,TYR249,HIS283,ALYS337,ASN338	ILE211,VAL219,ALA230,LEU260,LEU340,ALA350
W8	112.94	-7.73	2.16	LYS232,TYR249,HIS283,ASP351,ASN338	ILE211,VAL219,ALA230,LEU278,LEU340,ALA350
S1W8	124.65	-8.74	392.23	HIS283,LYS337,ASN338,APS351	ILE211,VAL219,ALA230,LEU260,LEU340,ALA350
BMS22	113.32	-9.25	165.85	LYS232,TYR249,HIS283,ASP351	ILE211,VAL219,ALA230,LEU278,LEU340,ALA350

Table S3-2. The chemical structures of 30 modified molecules

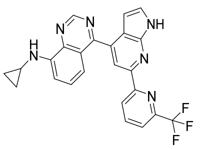 H1	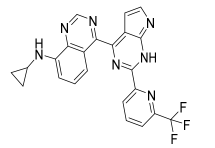 H2	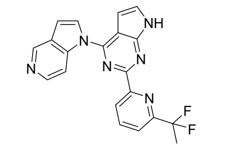 H3	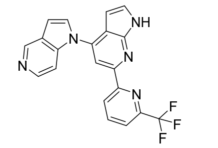 H4	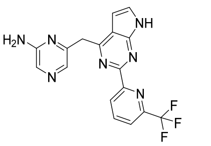 H5	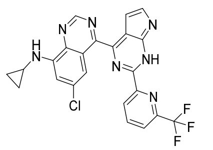 H6
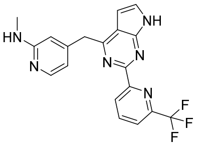 H7	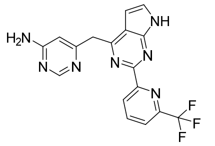 H8	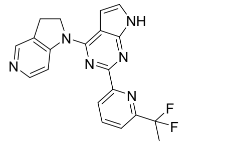 H9	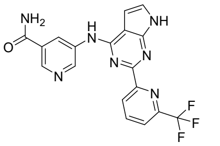 H10	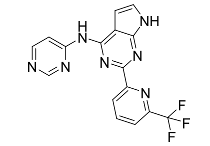 H11	
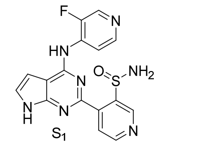 S1	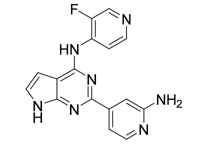 S2	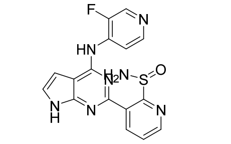 S3	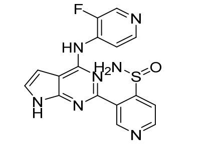 S4	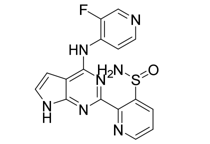 S5	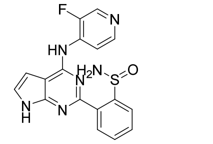 S6
S7 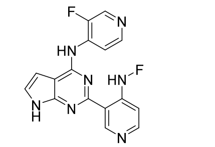	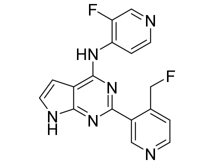 S8	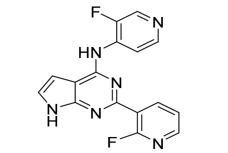 S9	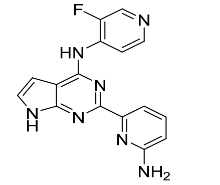 S10	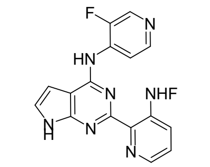 S11	
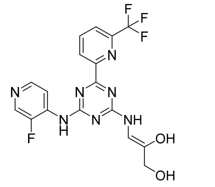 W1	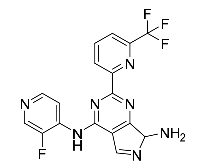 W2	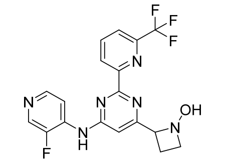 W3	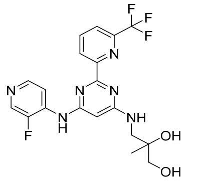 W4	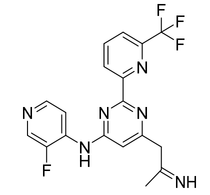 W5	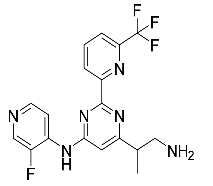 W6
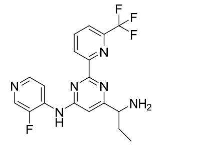 W7	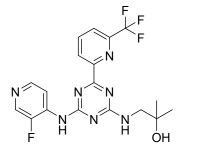 W8	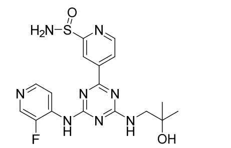 S1W8			

Figure S1. The chemical structures of modified molecules from hinge region (in red), Solvent channel region (in blue), and Selective pocket region (in yellow).

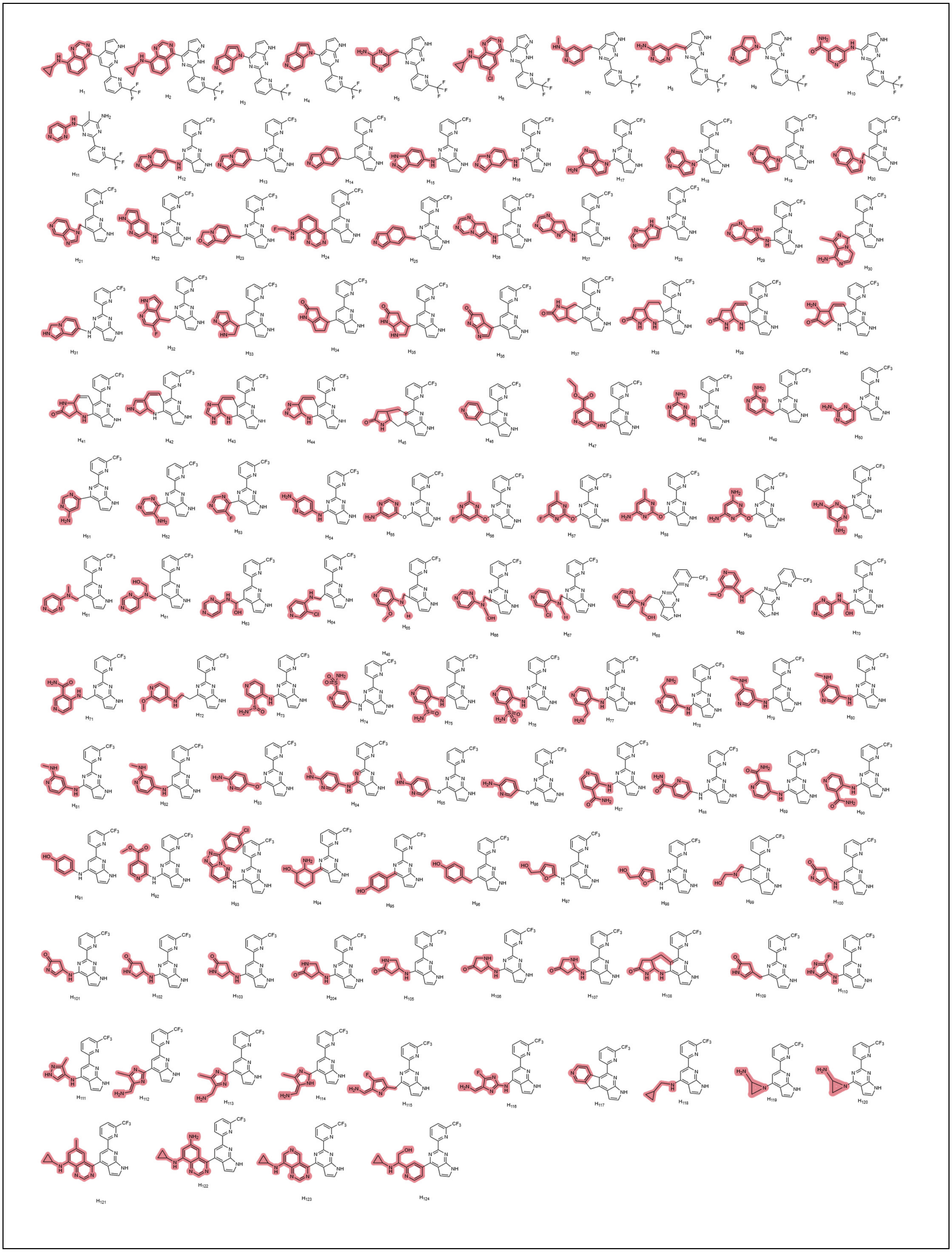

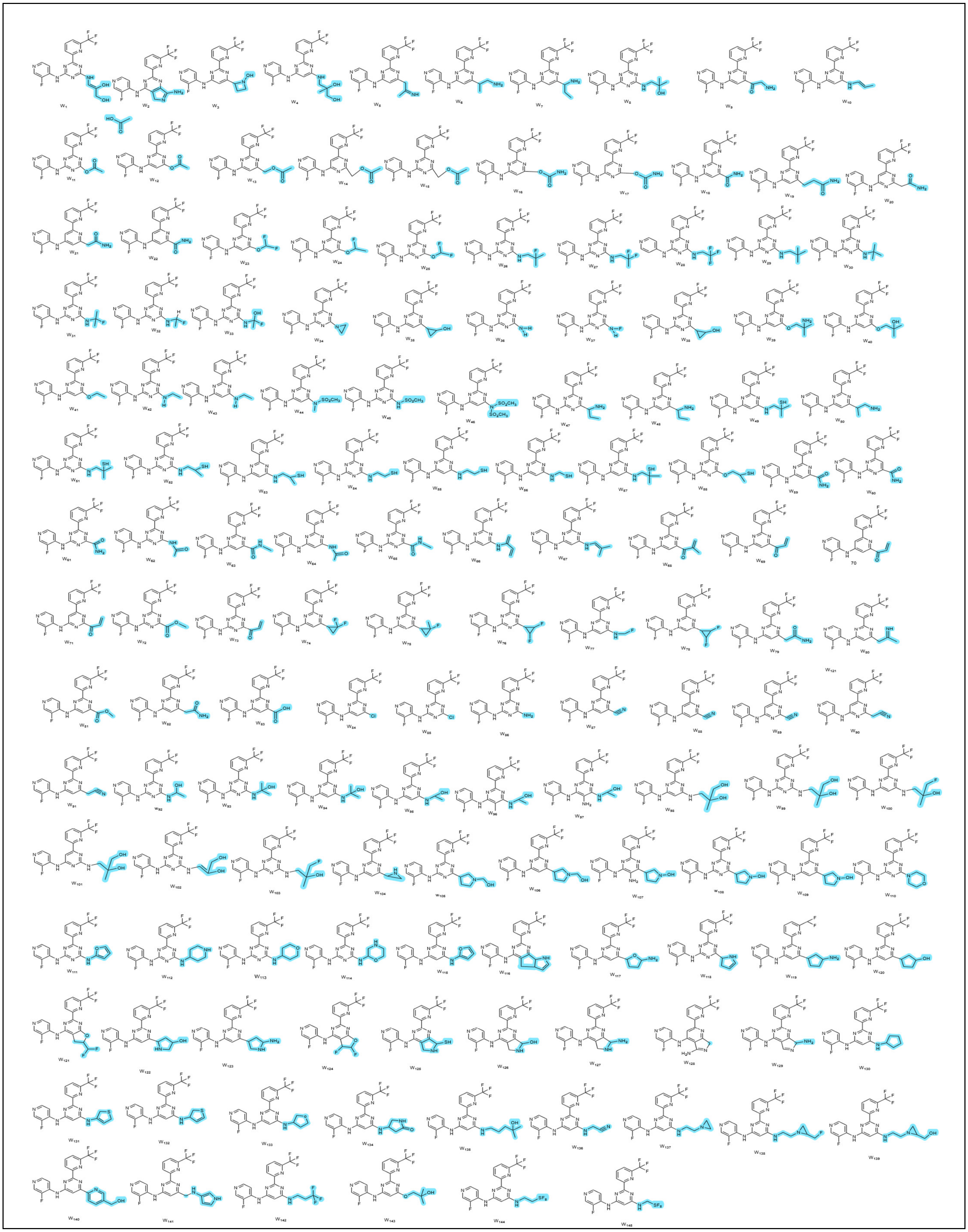

W1
W2
W3
W4
W5
W6
W7
W8
W9
W10
W11
W12
W13
W14
W15
W16
W17
W18
W19
W20
W21
W22
W23
W24
W25
W26
W27
W28
W29
W30
W31
W32
W33
W34
W35
W36
W37
W38
W39
W40
W41
W42
W43
W44
W45
W46
W47
W48
W49
W50
W51
W52
W53
W54
W55
W56
W57
W58
W59
W60
W61
W62
W63
W64
W65
W66
W67
W68
W69
70
W71
W72
W73
W74
W75
W76
W77
W78
W79
W80
W121
W81
W82
W83
W84
W85
W86
W87
W88
W89
W90
W91
W92
W93
W94
W95
W96
W97
W98
W99
W100
W101
W102
W103
W104
W105
W106
W107
W108
W109
W110
W111
W112
W113
W114
W115
W116
W117
W118
W119
W120
W121
W122
W123
W124
W125
W126
W127
W128
W129
W130
W131
W132
W133
W134
W135
W136
W137
W138
W139
W140
W141
W142
W143
W144
W145

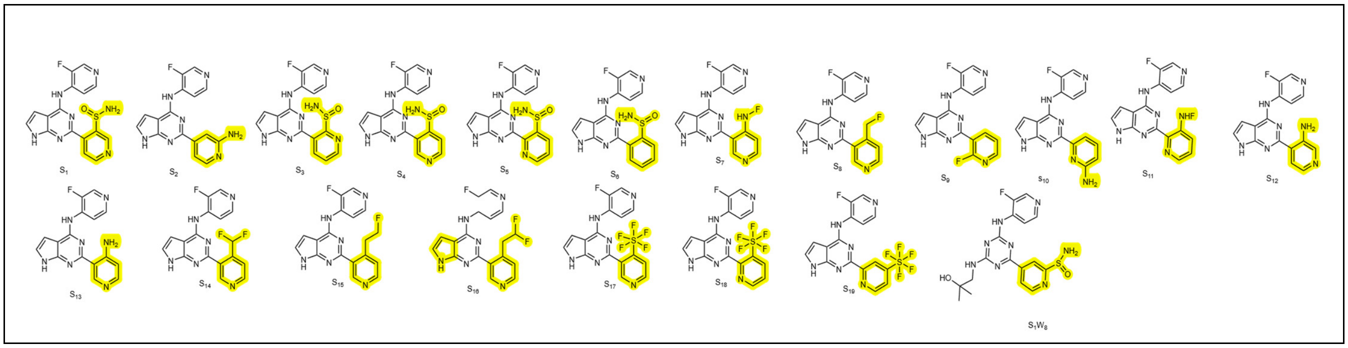

Figure S2.3D interaction pattern diagram between the protein and modified molecules in the crystal structure of 6B8Y.

Hinge region modified

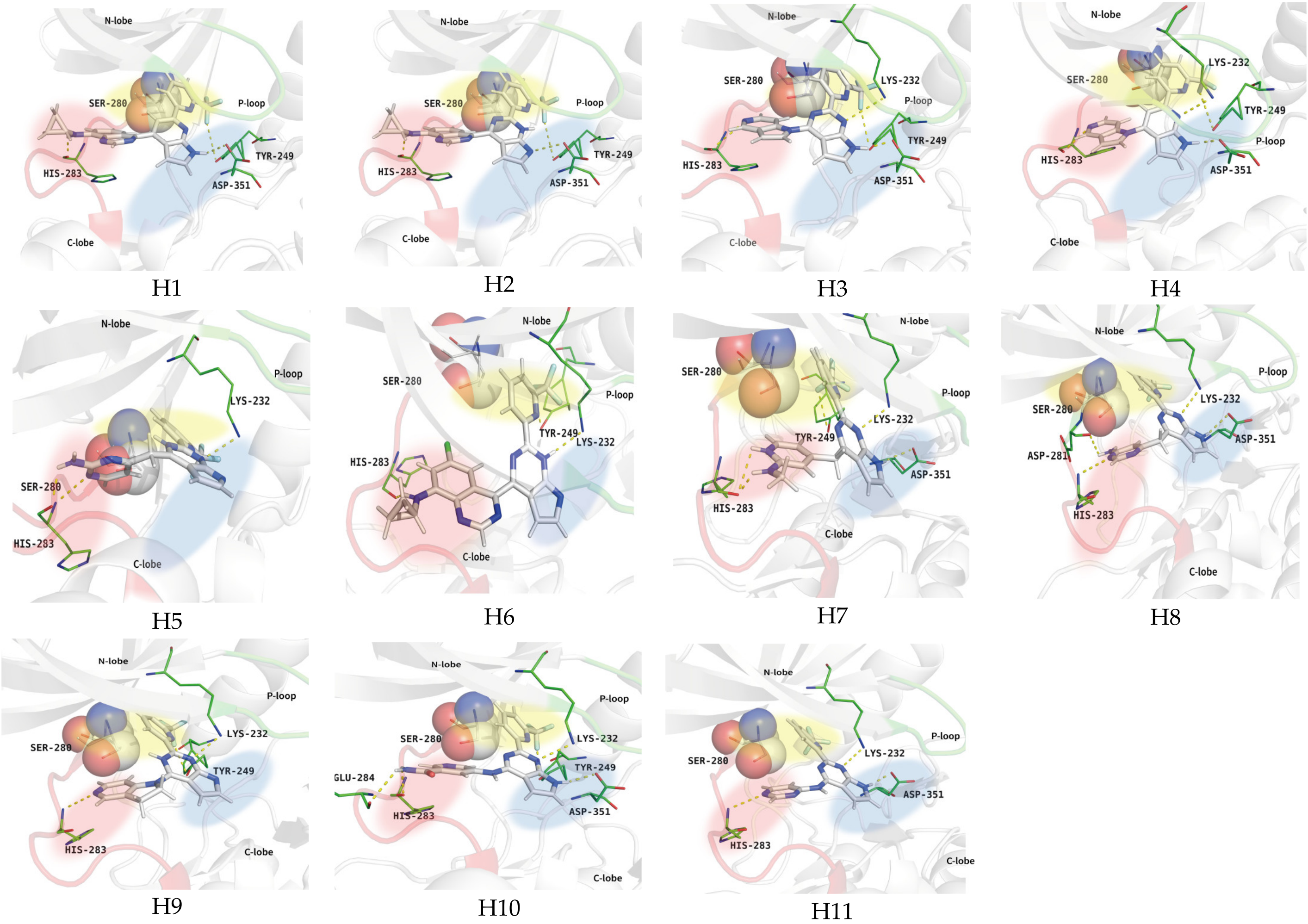

Selective pocket region modified:

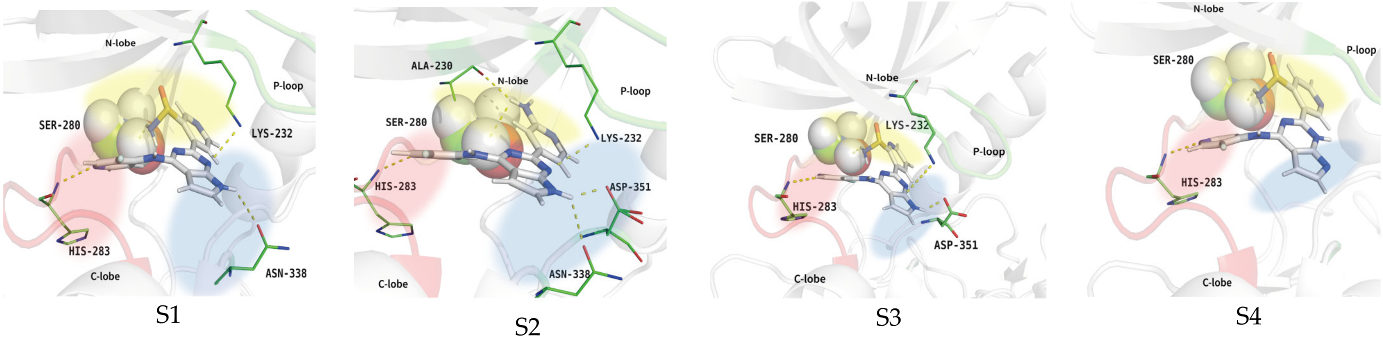

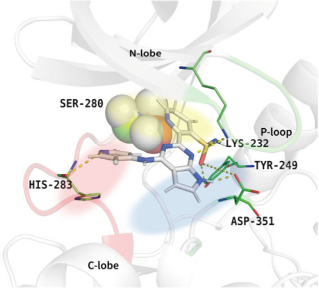

S5

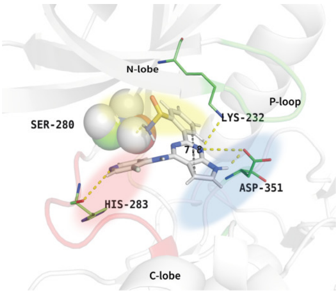

S6

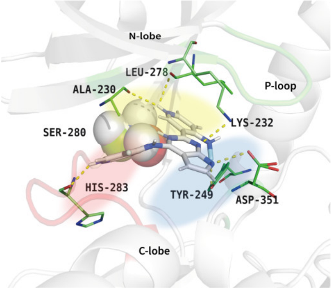

S7

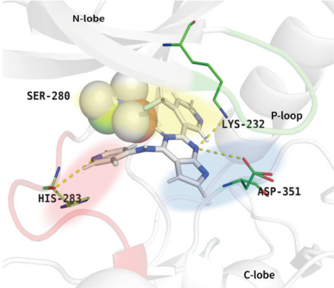

S8

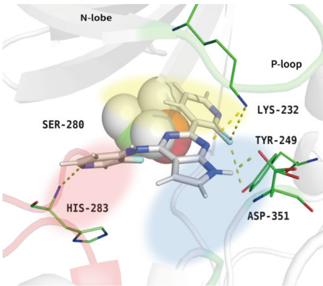

S9

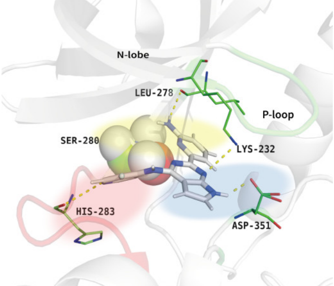

S10

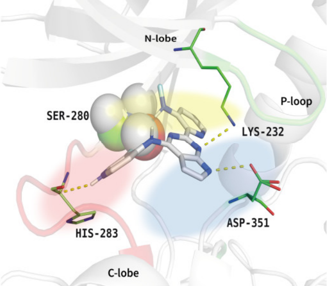

S11

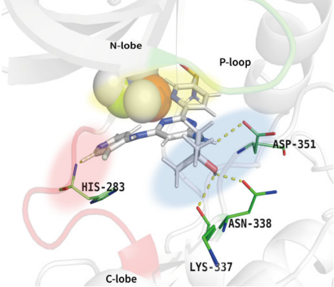

S1W8

Solvent channel region modified:

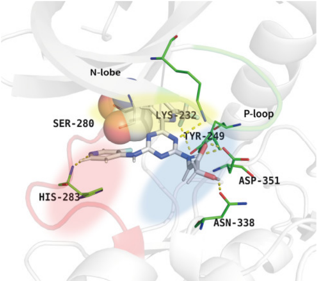

W1

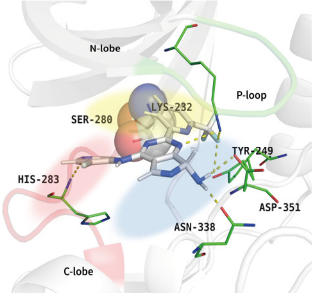

W2

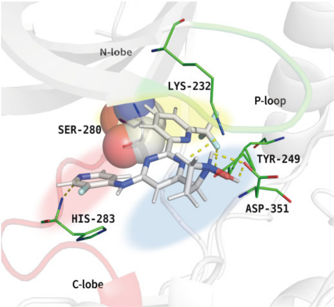

W3

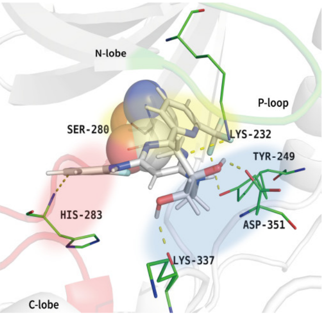

W4

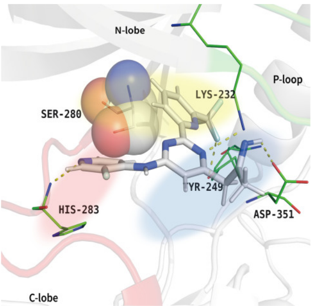

W5

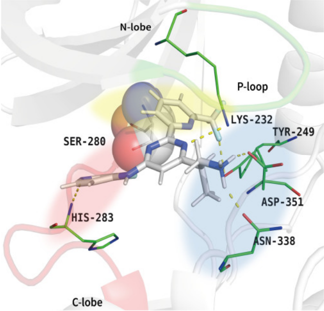

W6

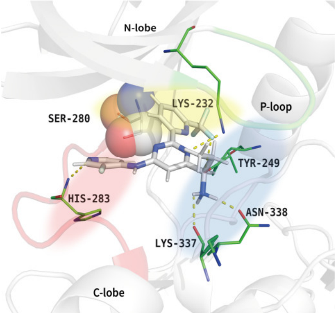

W7

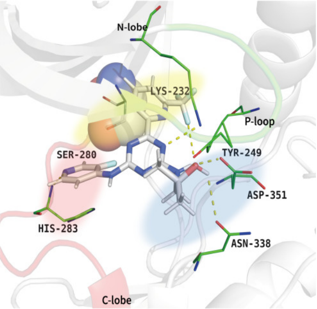

W8

Figure S3. Number of hydrogen bonds of five designed molecules and reference ligand BMS22 combined with TGFβRIduring 100 ns MD simulations.

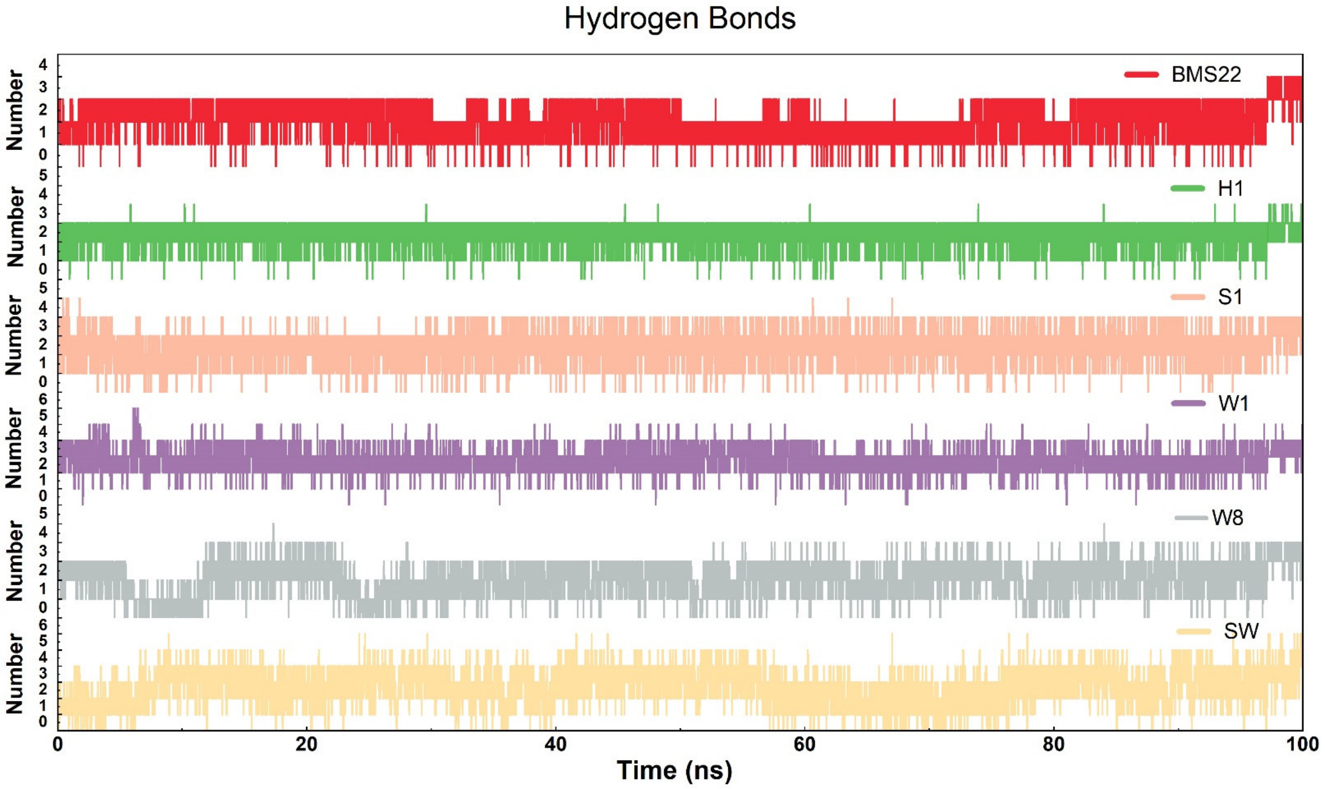

Table S4. Occupancy rates of hydrogen bonds between TGFβRI and each potential inhibitor.

	acceptor	donor H	donor	frac %[e]
BMS22	LIG@N15	HIS_283@H	HIS_283@N[a]	88.38%
	ASP_351@OD1b	LIG@H30	LIG@N9	58.87%
	LIG@N3	LYS@HZ2	LYS_232@NZ	32.03%
	ASP_351@OD2b	LIG@H30	LIG@N9	22.88%
	LIG@N23	LYS_232@HZ2	LYS_232@NZ	2.81%
H1	LIG@N1	LYS_232@HZ1	LYS_232@NZ	75.70%
	ASP_351@OD1[b]	LIG@H16	LIG@N5	61.22%
	HIS_283@O[a]	LIG@H15	LIG@N4	59.55%
	ASP_351@OD2[b]	LIG@H16	LIG@N5	33.40%
	LIG@N	LYS_232@HZ1	LYS_232@NZ	1.64%
S1	LIG@N5	HIS_283@H[a]	HIS_283@N[a]	81.43%
	ASP_351@OD2[b]	LIG@H7	LIG@N3	78.18%
	SER_280@OG[c]	LIG@H10	LIG@N6	53.47%
	ASP_281@O[a]	LIG@H11	LIG@N6	18.90%
	ALA_230@O	LIG@H11	LIG@N6	3.36%
W1	ASP_351@OD1[b]	LIG@H42	LIG@O28	94.67%
	ASP_351@OD1[b]	LIG@H41	LIG@N25	88.79%
	LIG@N21	HIS_283@H[a]	HIS_283@N[a]	80.00%
	LYS_213@O[d]	LIG@H43	LIG@O30	7.57%
	LIG@O28	ASN_338@HD21	ASN_338@ND2[b]	3.44%
W8	LIG@N7	HIS_283@H[a]	HIS_283@N[a]	83.97%
	ASP_351@OD2[b]	LIG@H16	LIG@O	49.02%
	ASP_351@OD2[b]	LIG@H14	LIG@N5	26.66%
	ASP_351@OD1[b]	LIG@H16	LIG@O	22.27%
	ASP_351@OD1[b]	LIG@H14	LIG@N5	20.47%
	LIG@O	ASN_338@HD22	ASN_338@ND2[b]	4.35%
S1W8	LIG@N4	HIS_283@H[a]	HIS_283@N[a]	85.11%
	LIG@O1	SER_280@HG1[a]	SER_280@OG[c]	51.60%
	ASP_351@OD1[b]	LIG@H17	LIG@O	38.91%
	ASP_351@OD1[b]	LIG@H16	LIG@N5	36.68%
	ASP_351@OD2[b]	LIG@ H16	LIG@N5	18.98%
	ASP_351@OD2[b]	LIG@H17	LIG@O	12.61%
	SER280@OG[c]	LIG@H18	LIG@N6	10.07%
	LYS_213@O[d]	LIG@H17	LIG@O	9.40%
	LIG@N5	ASN_338@HD22	ASN_338@ND2[b]	3.39%

a. Hydrogen bond interaction with the hinge region;
b. Hydrogen bond interaction with the solvent channel;
c. Hydrogen bond interaction with the selective hydrophobic pocket;
d. Hydrogen bond interaction with the P-loop region;
e. The hydrogen bonds are determined by the default criteria (acceptor··· H-donor angle of >120° and acceptor ··· donor distance of <3.5 Å) in the bonds.tcl of the VMD program

NMR spectra of synthesized compounds

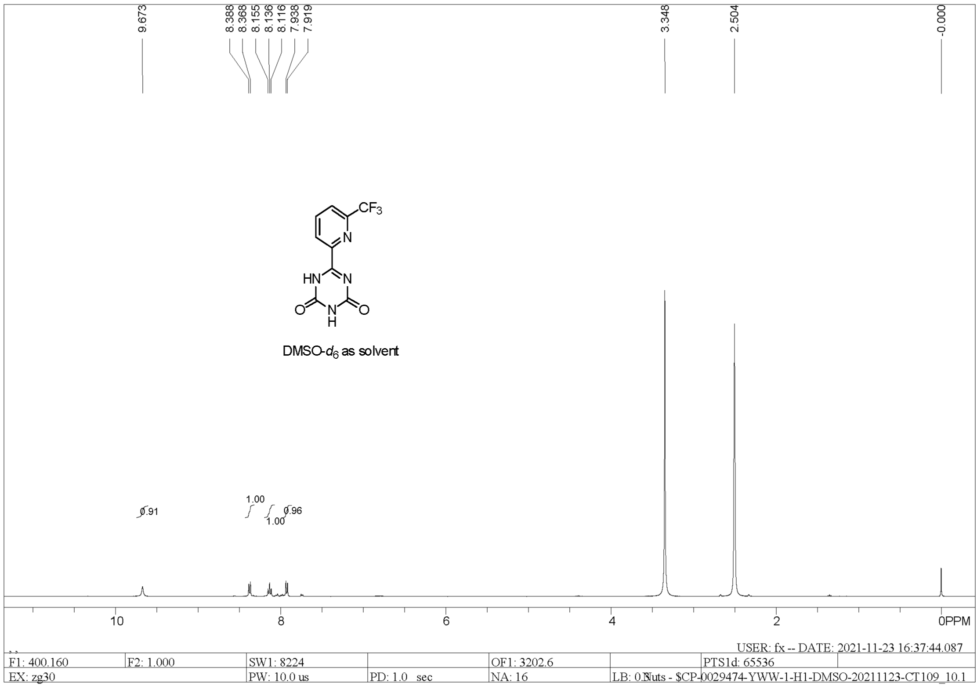

Figure S4 ^{1}H-NMR spectrum of 6-(6-(trifluoromethyl)pyridin-2-yl)-1,3,5-triazine-2,4(1H,3H)-dione.

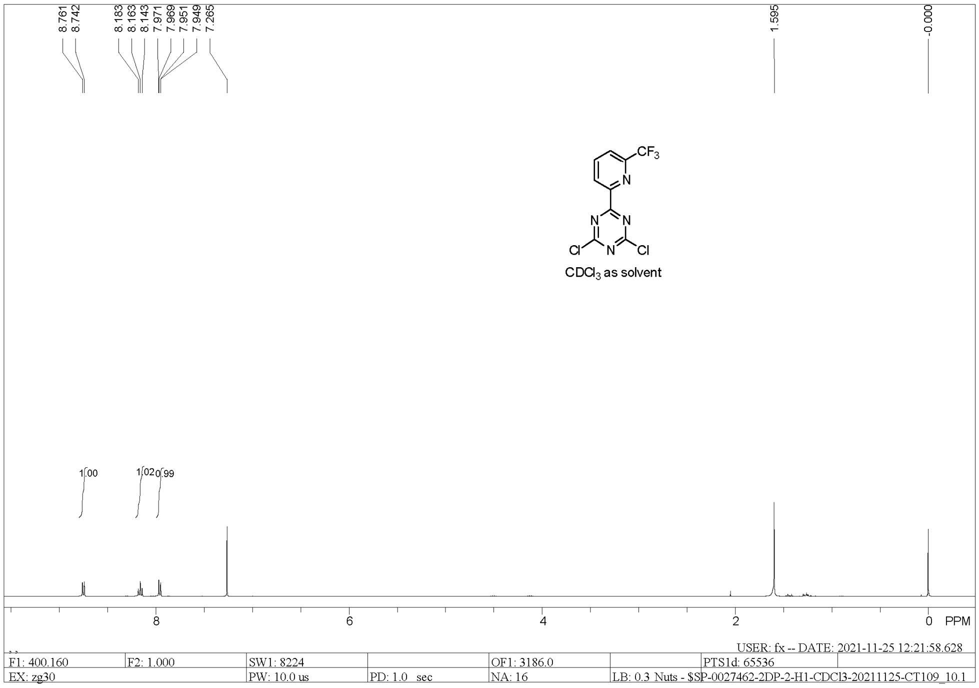

Figure S5 ^{1}H-NMR spectrum of 2,4-dichloro-6-(6-(trifluoromethyl)pyridin-2-yl)-1,3,5-triazine.

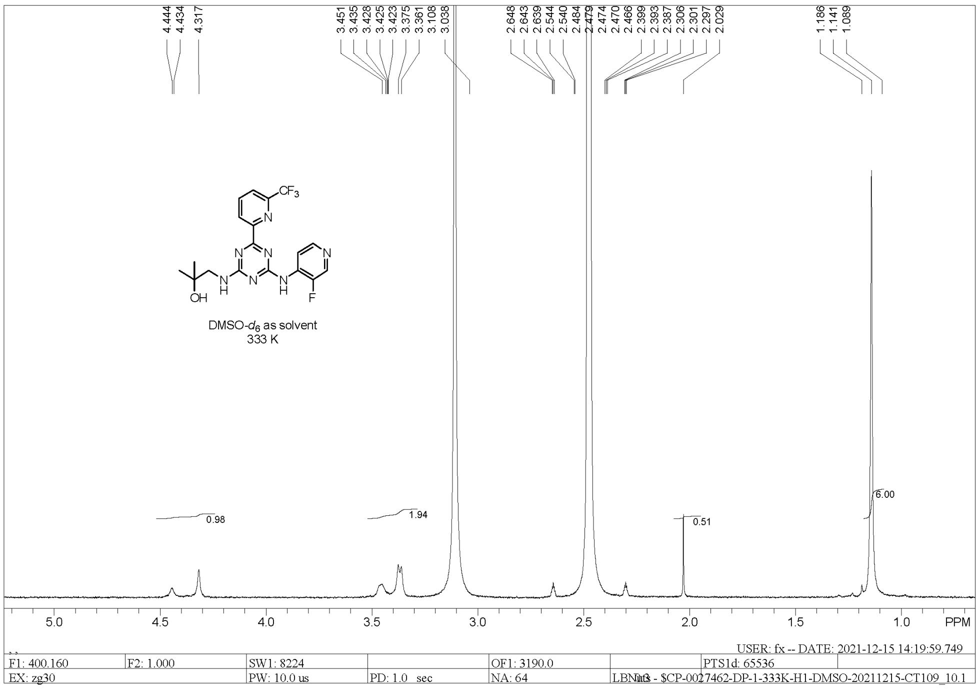

Figure S6 ^{1}H-NMR spectrum of 1-((4-((3-fluoropyridin-4-yl)amino)-6-(6-(trifluoromethyl)pyridin-2-yl)-1,3,5-triazin-2-yl)amino)-2-methylpropan-2-ol.

MS report 1： 6-(6-(trifluoromethyl)pyridin-2-yl)-1,3,5-triazine-2,4(1H,3H)-dione

```
File ..T109\MONITOR\SP-0027462-1P1-01831-LCMS047.D Tgt Mass (EZX): 258.00
Injection Date    : 23 Nov 21   5:32 pm +0800          Seq. Line : 0
Sample Name       : SP-0027462-1P1                  Location   : P1-F-04
Acq. Operator     : 109monitor                                Inj : 1
Spec. Reported    : MS Integration                     Inj Volume : 0.5 ul
Acq. Method       : C:\Chem32\1\METHODS\MONITOR 3MIN-50-B-P
Analysis Method   : C:\Chem32\1\METHODS\MONITOR 3MIN-50-B-P.M
Sample Info : Easy-Access Method: 'MONITOR 3MIN-50-B-P.M'   258.00
Method Info : Column: Waters X Bridge C18: 50mm*4.6 mm*3.5 um;
              Mobile Phase:  A: Water (0.01mol/L NH4HCO3)   B: ACN
              Gradient : B from 5% to 95% for 1.6 min and hold 95% for 1.4 min;
              Flow rate: 2.0 ml/min;
              Column Temperature: 40 ℃
```

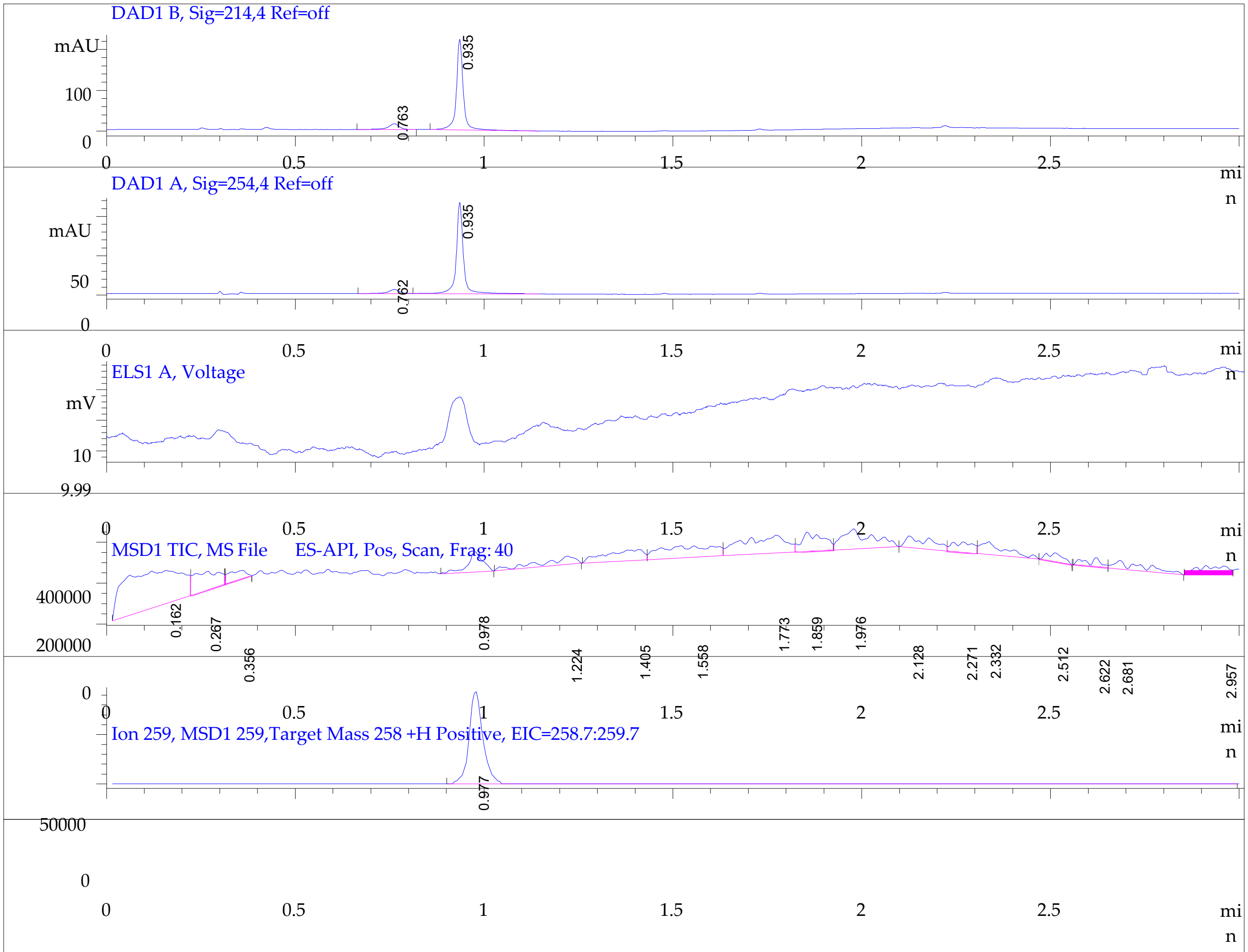

Integration Results for DAD1 B, Sig=214,4 Ref=off

RetTim	Width	Area	Height	Area%
0.76	0.03	29.00	13.86	9.45
0.94	0.02	277.97	221.62	90.55

Integration Results for DAD1 A, Sig=254,4 Ref=off

RetTim	Width	Area	Height	Area%
0.76	0.03	10.66	5.16	6.57
0.94	0.02	151.71	116.16	93.43

Integration Results for ELS1 A, Voltage

RetTim	Width	Area	Height	Area%

Integration Results for MSD1 TIC, MS File

RetTim	Width	Area	Height	Area%

0.16	0.15	1894002.38	156914.33	27.63
0.27	0.06	437014.69	89740.66	6.38
0.36	0.05	155703.45	41483.01	2.27
0.98	0.04	271918.50	95768.83	3.97
1.22	0.08	221813.67	37366.83	3.24
1.40	0.09	382647.88	53072.18	5.58
1.56	0.12	556172.44	55576.87	8.11
1.77	0.10	700590.00	86905.44	10.22
1.86	0.05	365538.19	89596.53	5.33
1.98	0.08	526599.06	92715.45	7.68
2.13	0.06	263161.16	54866.41	3.84
2.27	0.05	189155.39	51044.39	2.76
2.33	0.06	277677.00	58777.80	4.05
2.51	0.05	128105.84	43750.95	1.87
2.62	0.04	118888.68	45586.55	1.73
2.68	0.07	219188.27	40898.83	3.20
2.96	0.08	146532.42	24227.98	2.14

MS Report

Ret. Time: 0.16 <<<< POSITIVE SPECTRA >>>>

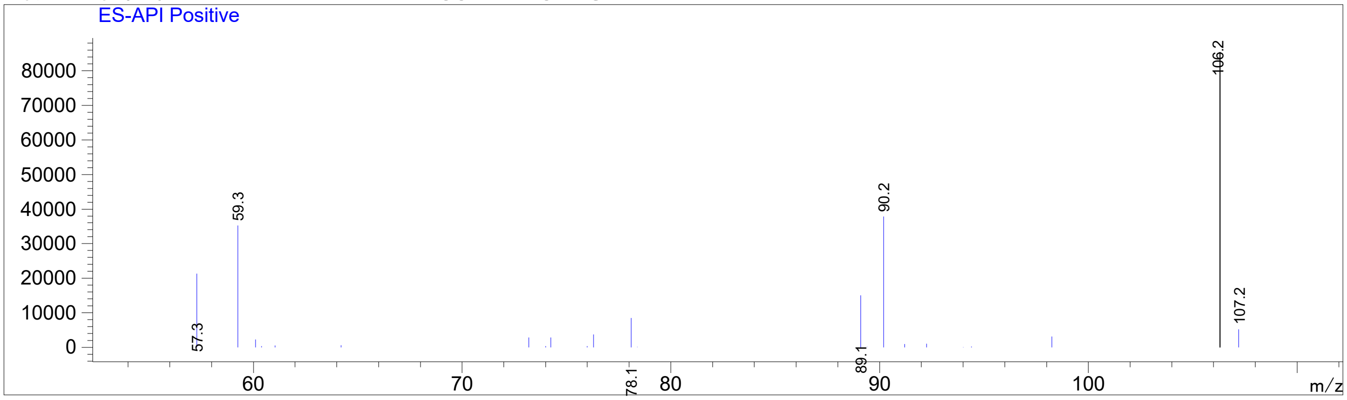

Ret. Time: 0.27

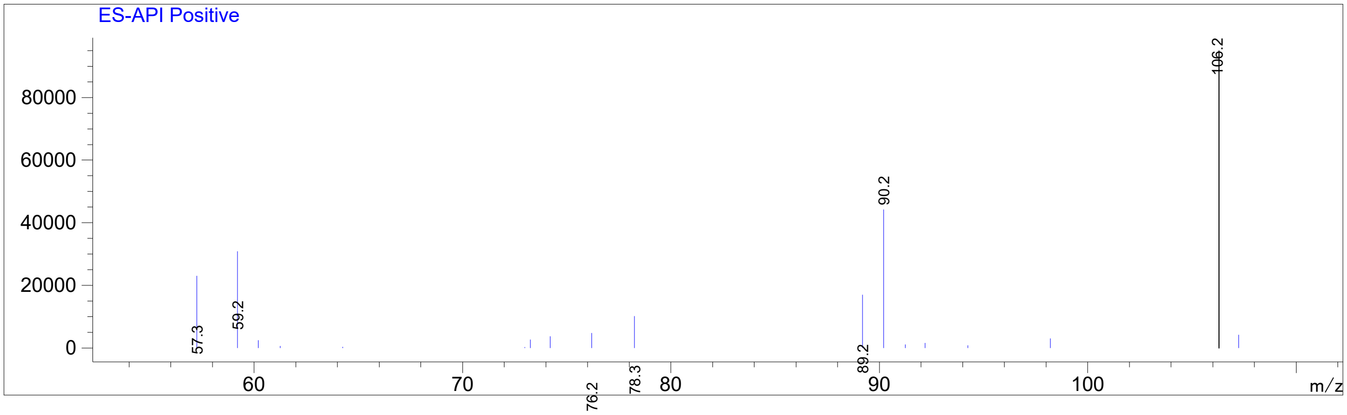

Ret. Time: 0.36

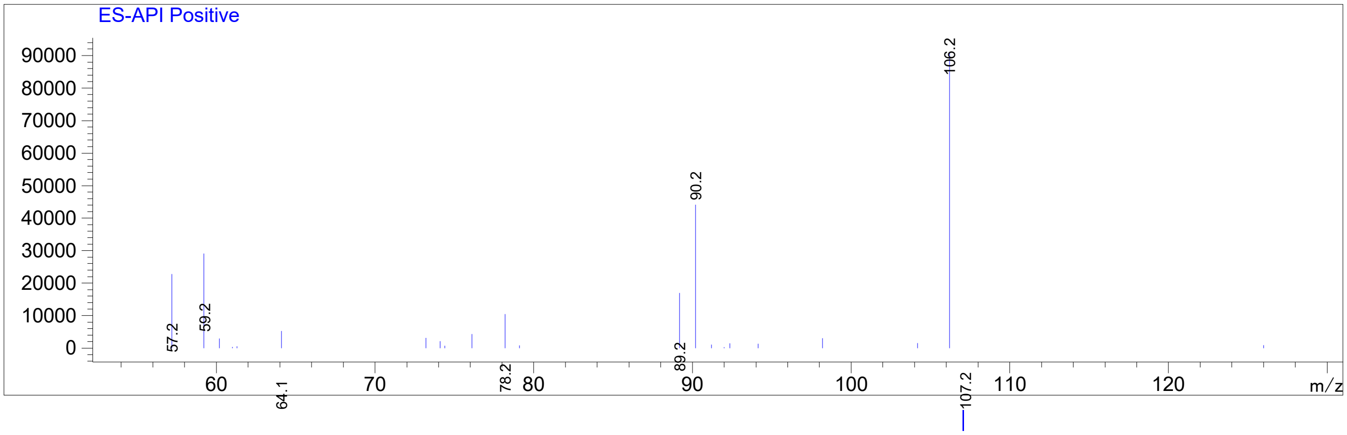

Ret. Time: 0.98

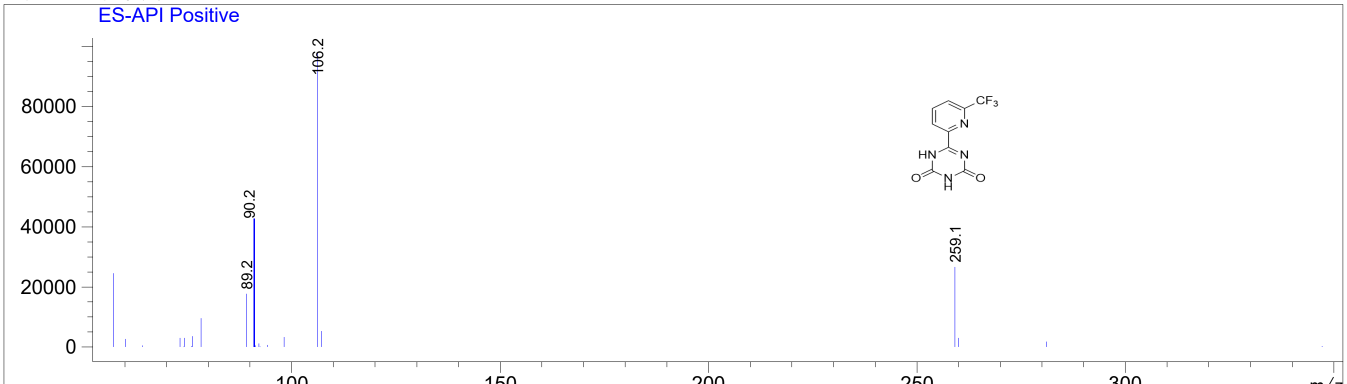

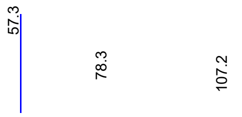
57.3
78.3
107.2

MS Report

Ret. Time: 1.22

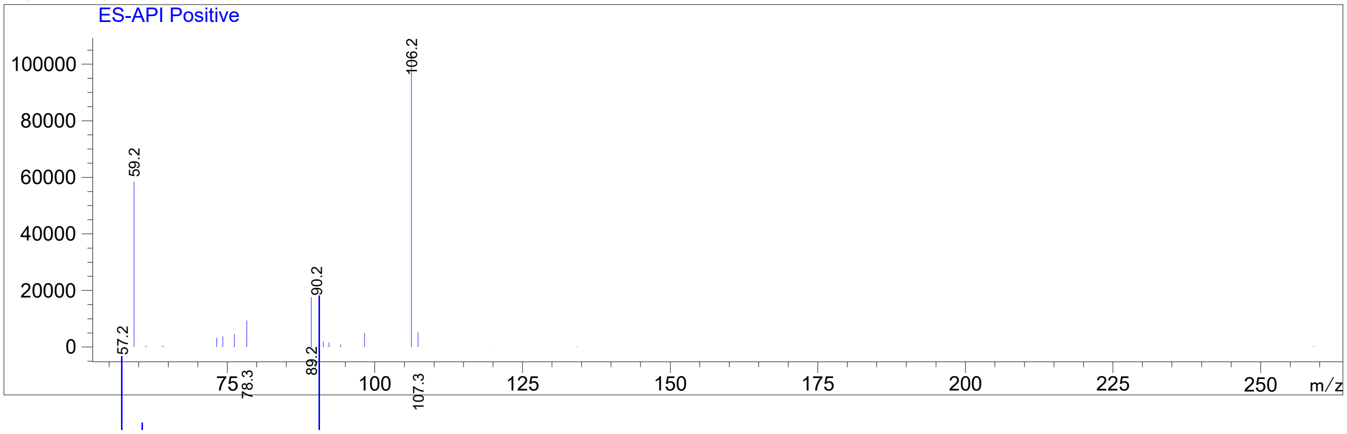

Ret. Time: 1.40

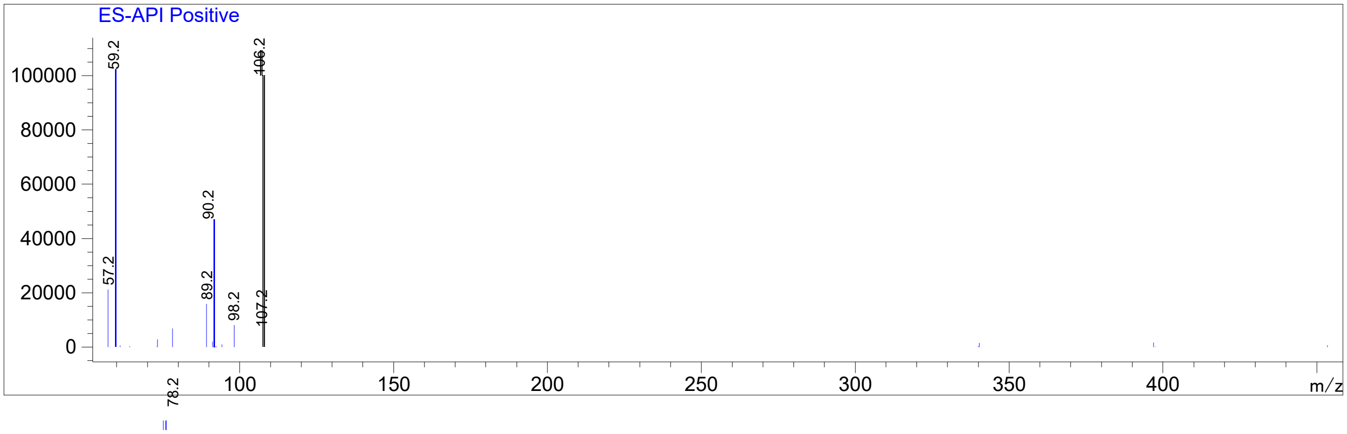

Ret. Time: 1.56

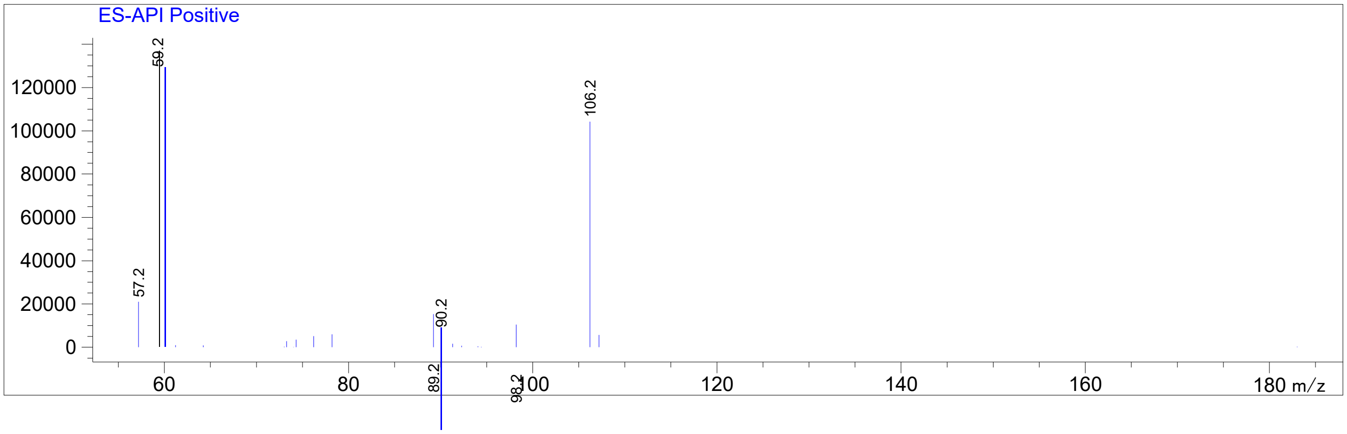

Ret. Time: 1.77

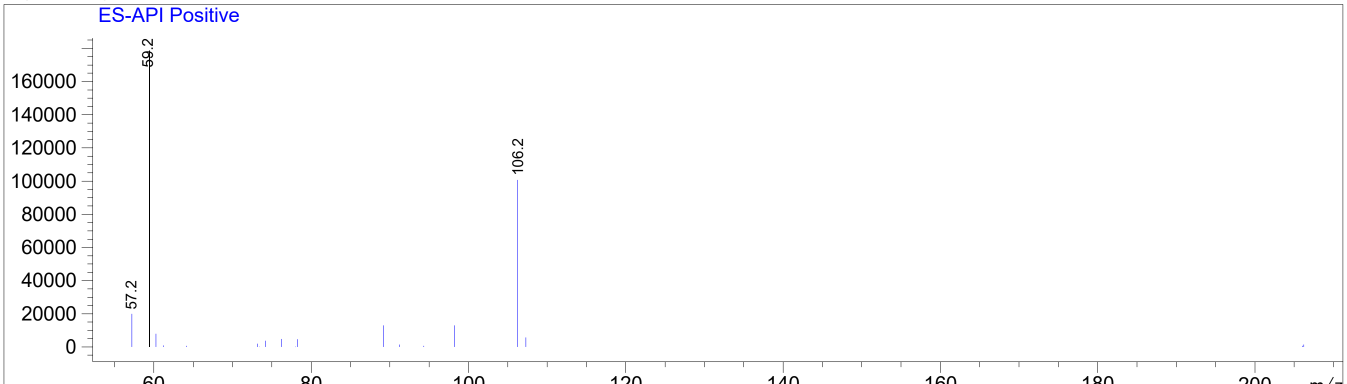

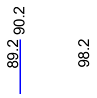
89.2
90.2
98.2

MS Report

Ret. Time: 1.86

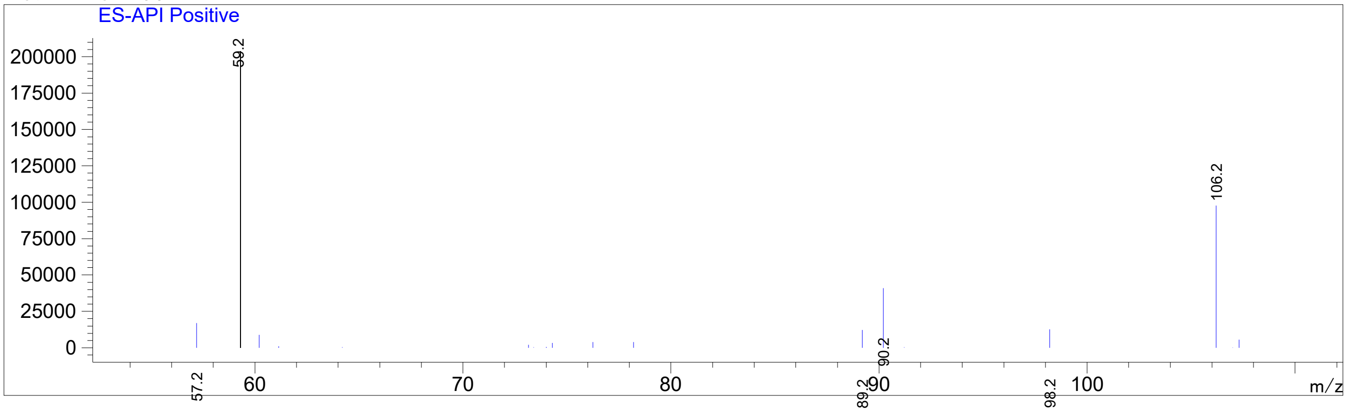

Ret. Time: 1.98

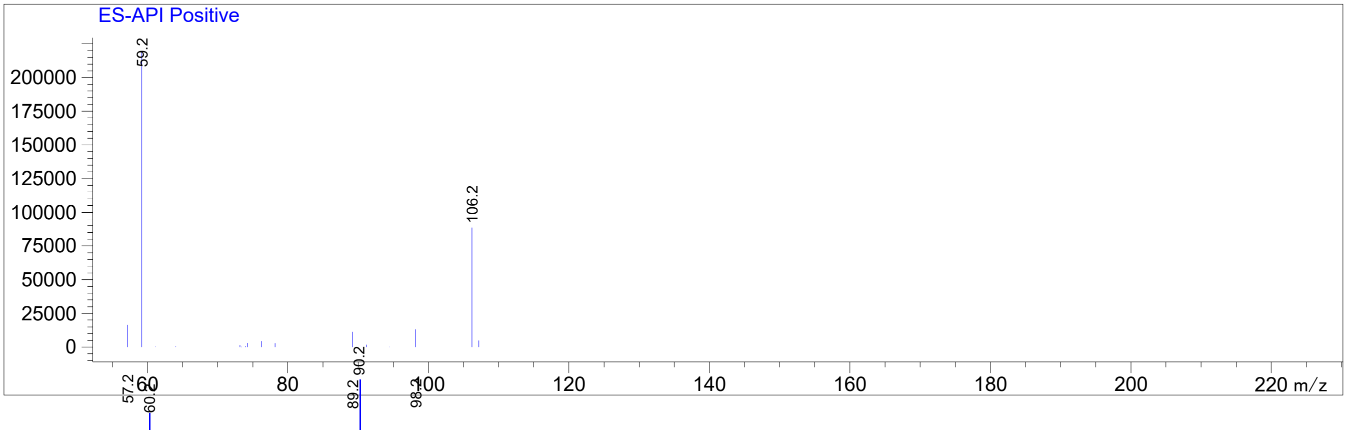

Ret. Time: 2.13

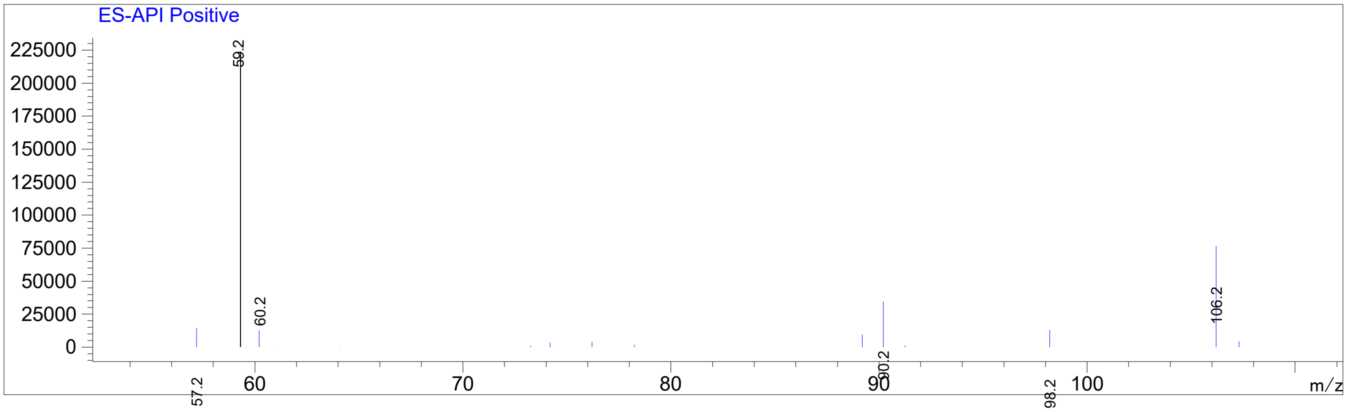

Ret. Time: 2.27

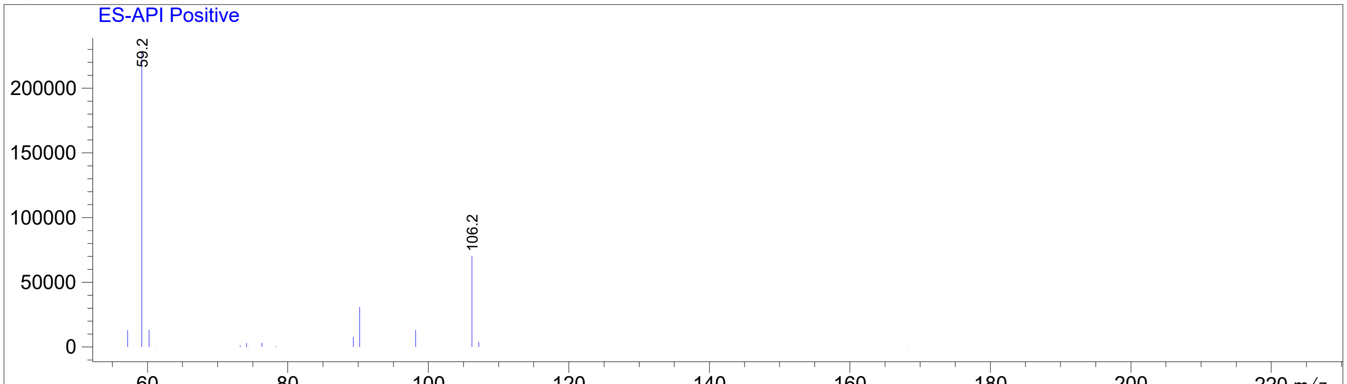

57.2
60.3
90.2
98.2

MS Report

Ret. Time: 2.33

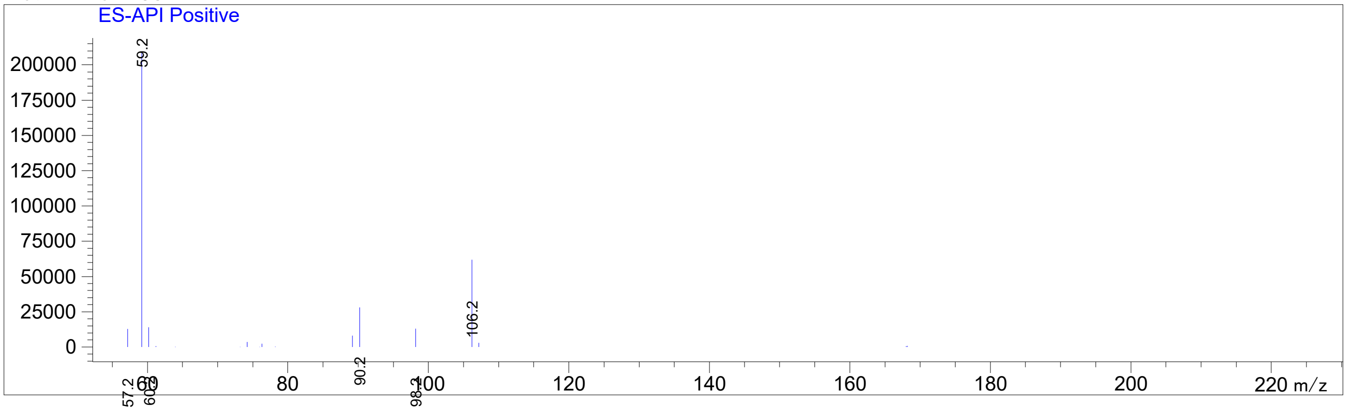

Ret. Time: 2.51

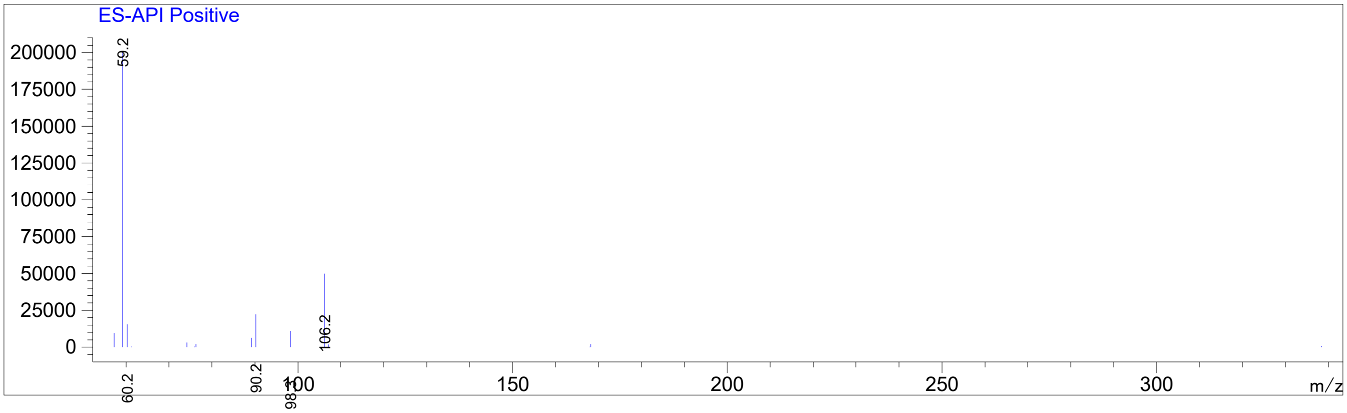

Ret. Time: 2.62

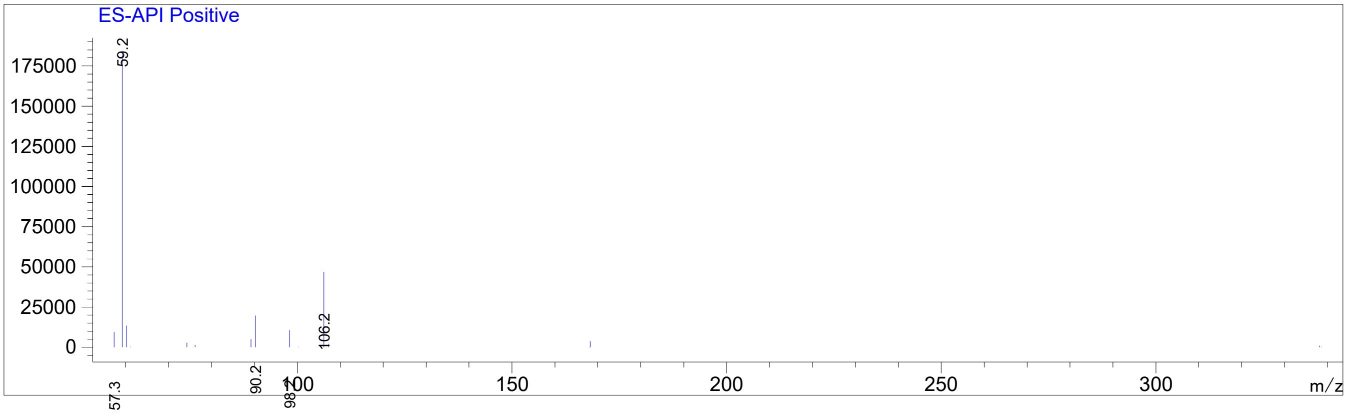

Ret. Time: 2.68

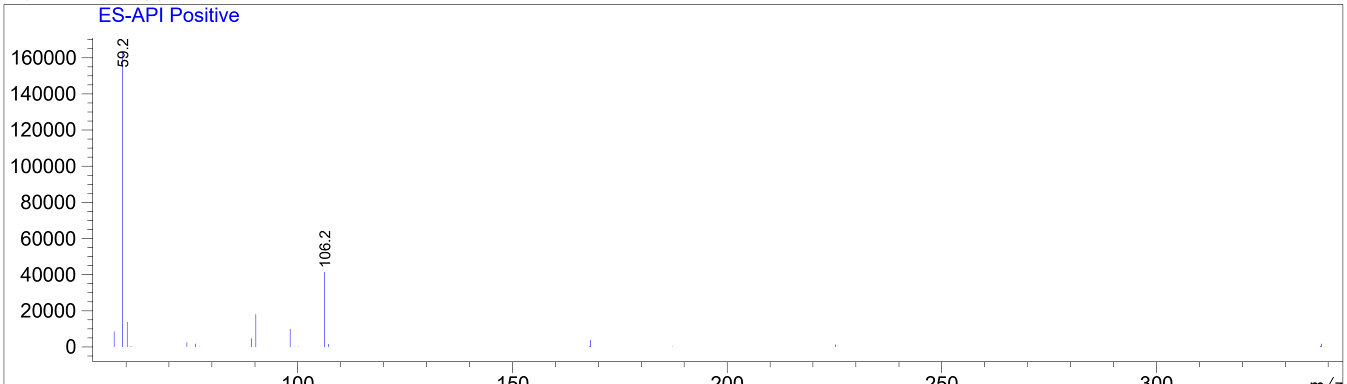

57.2

90.2

98.2

MS Report

Ret. Time: 2.96

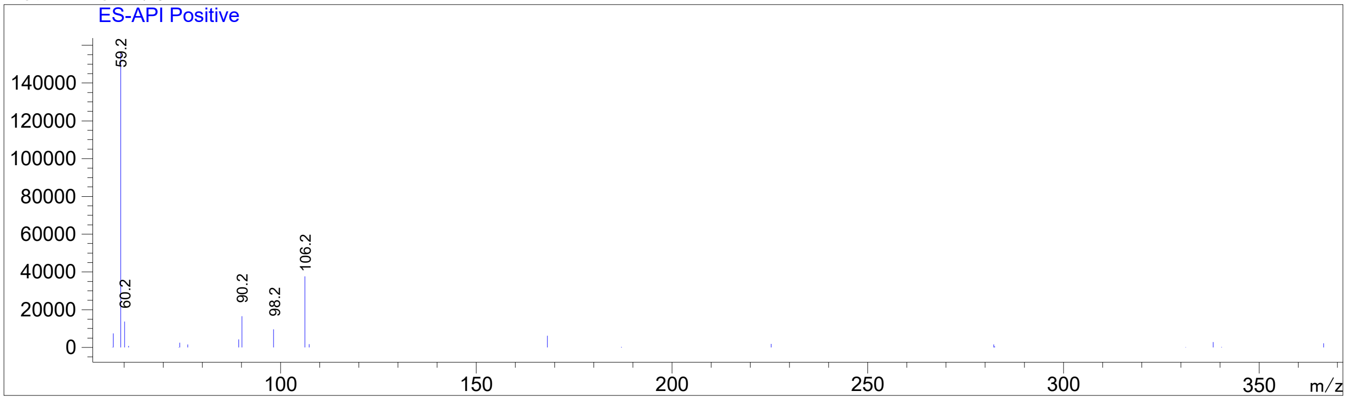

MS report 2： 2,4-dichloro-6-(6-(trifluoromethyl)pyridin-2-yl)-1,3,5-triazine

```
File ..09\MONITOR\SP-0027462-2P1-04381-LCMSA 046.D Tgt Mass (EZX): 294.00
Injection Date    : 25 Nov 21  11:06 am +0800          Seq. Line : 0
Sample Name       : SP-0027462-2P1                Location   : P2-F-08
Acq. Operator     : 109monitor                                Inj : 1
Spec. Reported    : MS Integration                      Inj Volume : 0.5 ul
Acq. Method       : C:\Chem32\1\METHODS\MONITOR 2.75MIN-B-P
Analysis Method   : C:\Chem32\1\METHODS\MONITOR 2.75MIN-B-P.M
Sample Info : Easy-Access Method: 'MONITOR 2.75MIN-B-P.M'   294.00
Method Info : Column: Phenomenex Kinetex EVO C18, 50mm*4.6 mm, 2.6 um
              Mobile Phase:  A: Water (0.01mol/L NH4HCO3)  B: ACN
              Gradient : 5-100% B (MeCN) in 1.75min, 100% at 2.55min, 5% at 2.65min,  en
              Flow rate: 2.3 ml/min
              Column Temperature: 40 ℃
```

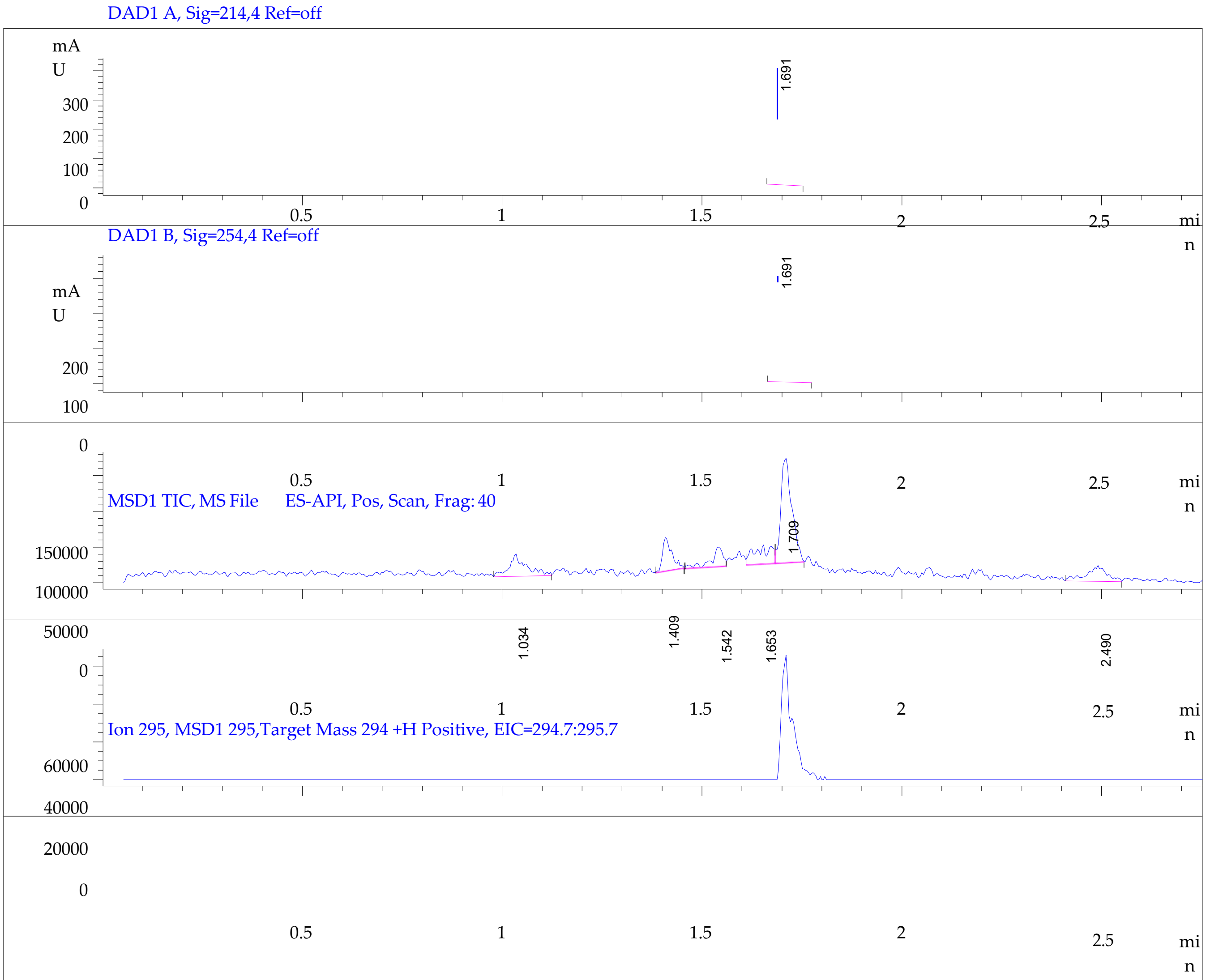

Integration Results for DAD1 A, Sig=214,4 Ref=off

RetTim	Width	Area	Height	Area%
1.69	0.01	394.73	412.01	100.00

Integration Results for DAD1 B, Sig=254,4 Ref=off

RetTim	Width	Area	Height	Area%
1.69	0.01	331.63	343.54	100.00

Integration Results for MSD1 TIC, MS File

RetTim	Width	Area	Height	Area%
1.03	0.04	91138.29	31642.16	13.82
1.41	0.02	76221.73	47303.34	11.55
1.54	0.03	56649.94	26844.77	8.59

1.65	0.04	79144.08	27141.74	12.00
1.71	0.03	281485.75	147665.81	42.67
2.49	0.04	75003.82	21971.37	11.37

MS Report

Ret. Time: 1.03 <<<< POSITIVE SPECTRA >>>>

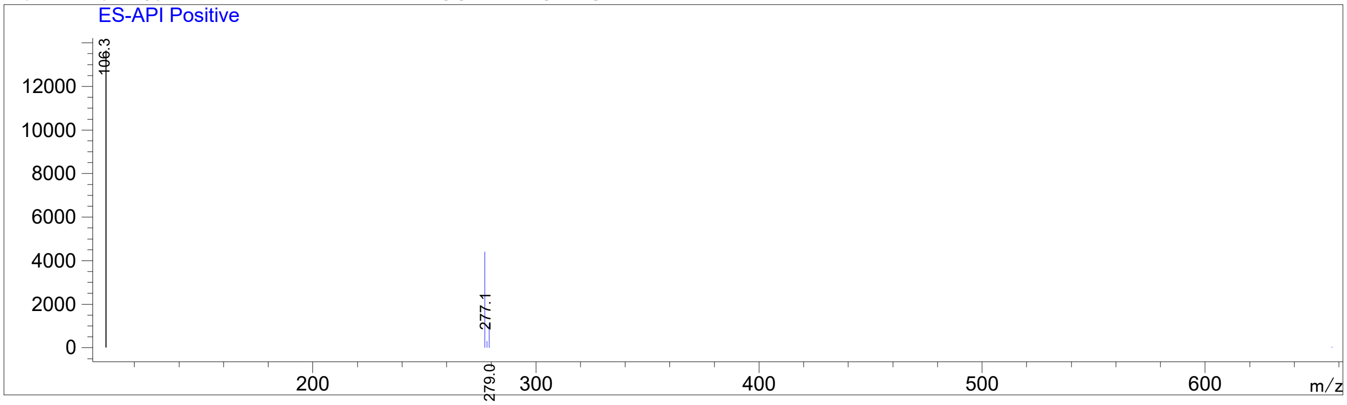

Ret. Time: 1.41

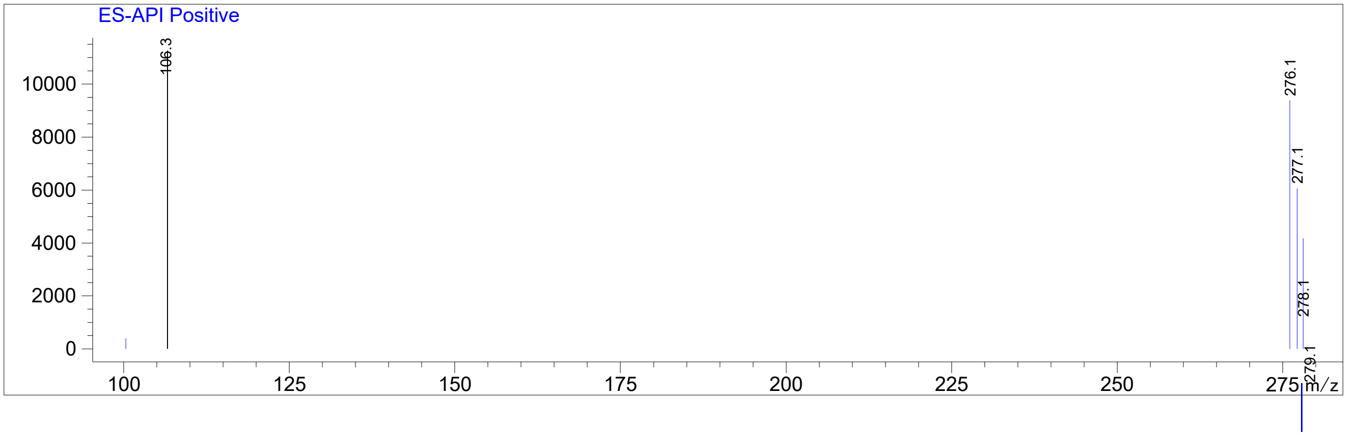

Ret. Time: 1.54

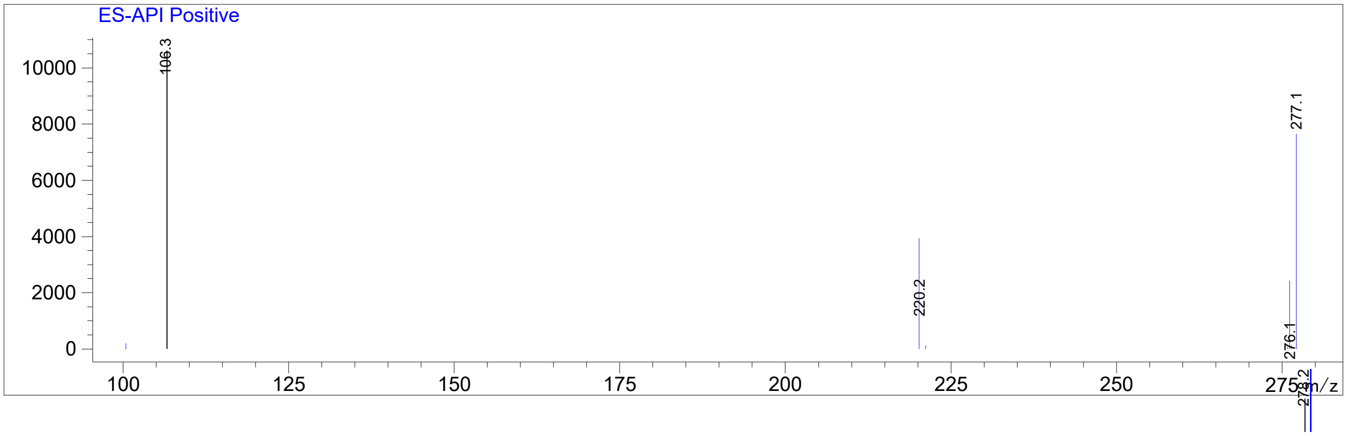

Ret. Time: 1.65

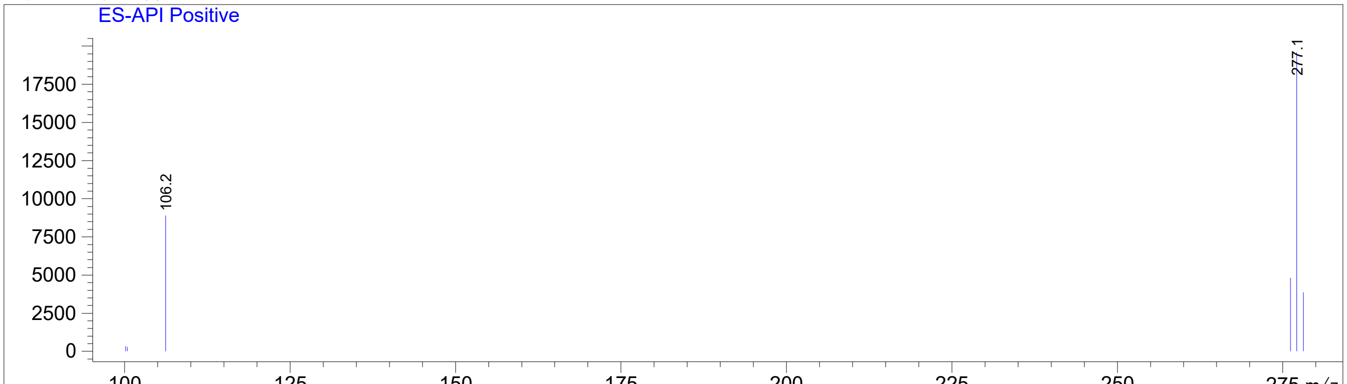

MS Report

Ret. Time: 1.71

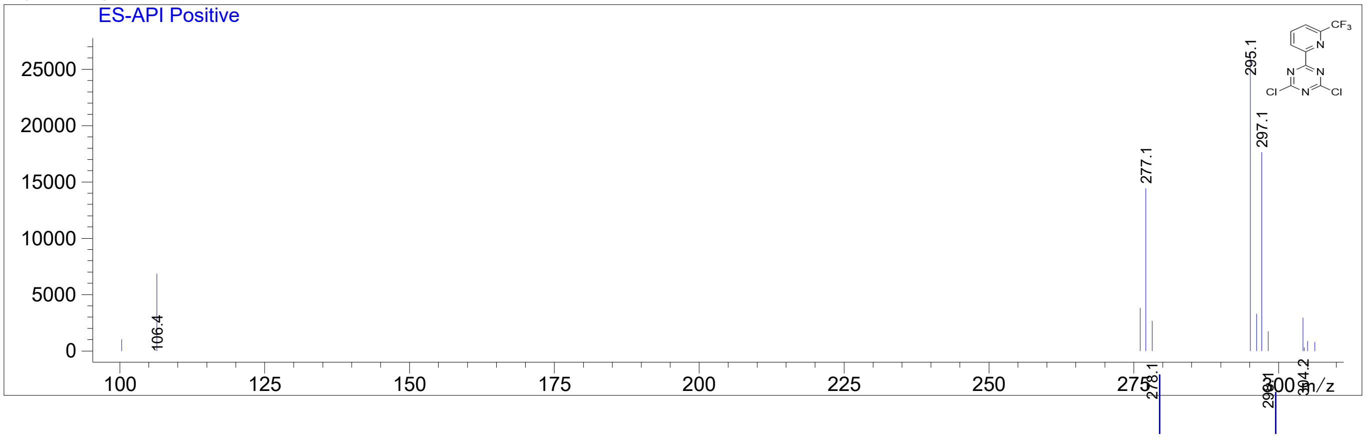

Ret. Time: 2.49

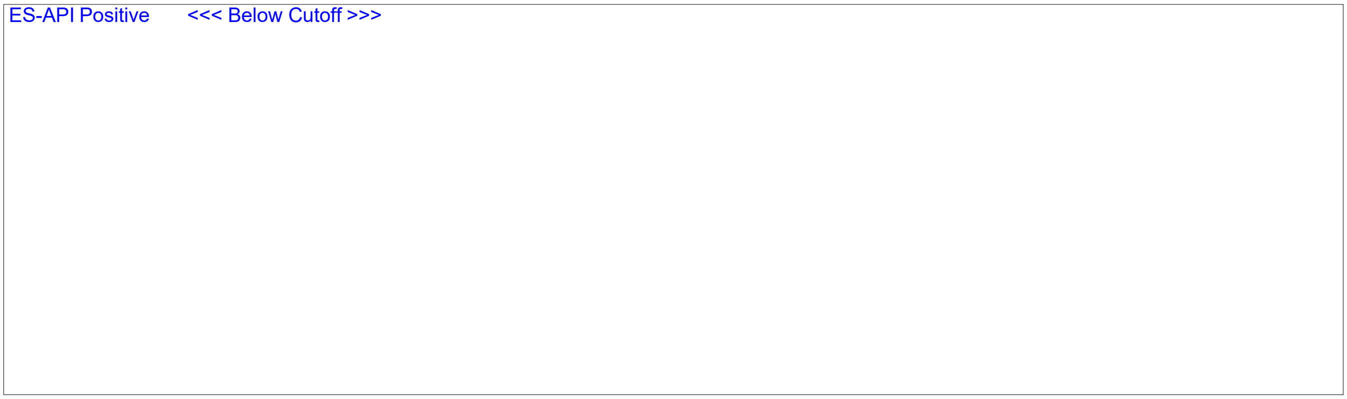

MS report 3: 1-((4-((3-fluoropyridin-4-yl)amino)-6-(6-(trifluoromethyl)pyridin-2-yl)-1,3,5- triazin-2-yl)amino)-2-methylpropan-2-ol

```
File ..T109\MONITOR\SP-002764-DP-1-03410-LCMS047.D Tgt Mass (EZX): 423.00
Injection Date    :  8 Dec 21   1:51 pm +0800          Seq. Line : 0
Sample Name       : SP-002764-DP-1                  Location   : P1-A-09
Acq. Operator     : 109monitor                                Inj : 1
Spec. Reported    : MS Integration                     Inj Volume : 3 ul
Acq. Method       : C:\Chem32\1\METHODS\MONITOR 3MIN-50-B-P
Analysis Method   : C:\CHEM32\1\METHODS\MONITOR 3MIN-B-P.M
Sample Info : Easy-Access Method: 'MONITOR 3MIN-50-B-P.M'   423.00
Method Info : Column: Waters X Bridge C18: 50mm*4.6 mm*3.5 um;
              Mobile Phase:  A: Water (0.01mol/L NH4HCO3)   B: ACN
              Gradient : B from 5% to 95% for 1.6 min and hold 95% for 1.4 min;
              Flow rate: 2.0 ml/min;
              Column Temperature: 40 ℃
```

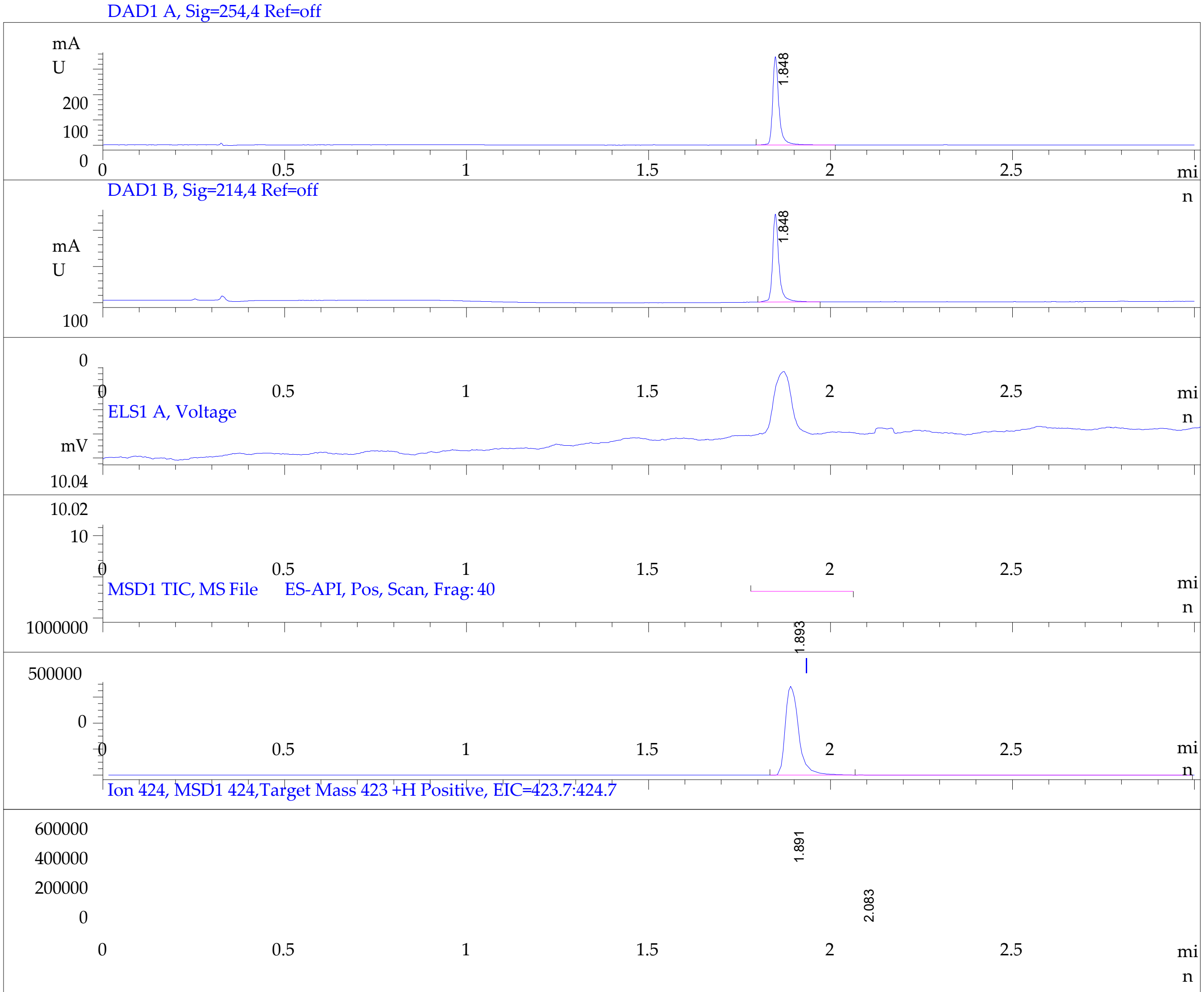

Integration Results for DAD1 A, Sig=254,4 Ref=off

RetTim	Width	Area	Height	Area%
1.85	0.02	413.09	348.04	100.00

Integration Results for DAD1 B, Sig=214,4 Ref=off

RetTim	Width	Area	Height	Area%
1.85	0.02	292.27	242.33	100.00

Integration Results for ELS1 A, Voltage

RetTim	Width	Area	Height	Area%

Integration Results for MSD1 TIC, MS File

RetTim	Width	Area	Height	Area%
1.89	0.04	2232784.50	765318.81	100.00

MS Report

Ret. Time: 1.89 <<<< POSITIVE SPECTRA >>>>

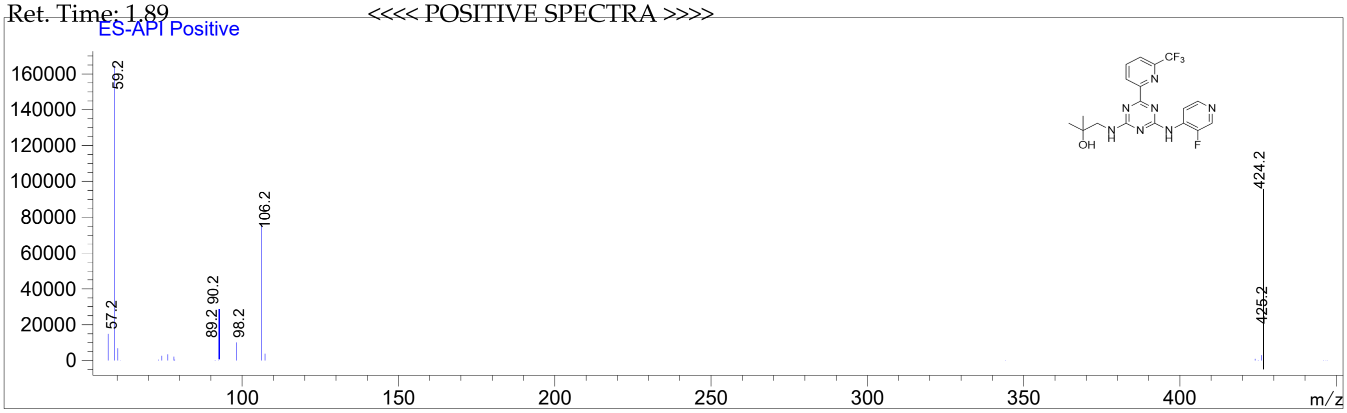

Supplement: Supplementary file 1 [file pharmaceuticals-15-01264-s001.zip › pharmaceuticals-1936266-supplementary.pdf]
